# The factor structure of the Zanarini Rating Scale for Borderline Personality Disorder: Exploratory Structural Equation Modelling and measurement invariance over time

**DOI:** 10.1002/mpr.1874

**Published:** 2021-05-12

**Authors:** Boliang Guo, Lingyan Li, Michael J. Crawford, Richard Morriss

**Affiliations:** ^1^ Division of Psychiatry and Applied Psychology Institute of Mental Health University of Nottingham London UK; ^2^ Department of Nursing Nanchang University Nanchang PR China; ^3^ Centre for Psychiatry Imperial College London Nottingham UK

**Keywords:** borderline personality disorder, factor structure, Exploratory Structural Equation Modelling, measurement invariance, ZAN‐BPD

## Abstract

**Objectives:**

There is a lack of independent longitudinal evidence on the factor structure and validity of the Zanarini Rating Scale for Borderline Personality Disorder (ZAN‐BPD). This study aimed to investigate the dimensionality of ZAN‐BPD and its conceptual consistency over time.

**Methods:**

Adult BPD participants (*n* = 276) were recruited for a multicentre, two‐arm randomised clinical trial with ZAN‐BPD measured at baseline and follow up at 12, 24 and 52 weeks. The construct and stability of the ZAN‐BPD across 52 weeks was examined through a measurement equivalence/invariance procedure via Exploratory Structural Equation Modelling.

**Results:**

Factor analysis results showed that the ZAN‐BPD had a bi‐2 factor structure that was stable over 52 weeks with a general factor and two specific factors. Factor loadings for eight of the nine items were greater for the general factor than the two specific factors. Factor 1 contrasts externalising distress with internalising distress. Factor 2 contrasts depression and self‐destruction with interpersonal anxiety and conflict.

**Conclusion:**

ZAN‐BPD is a conceptually and empirically valid measure of total BPD symptom severity in BPD patients over time suitable for use in clinical trials. Two factors related to the expression of distress and self‐harm may be utilised as possible predictors of outcome.

## 1 INTRODUCTION

Borderline personality disorder (BPD) is a serious psychiatric disorder, characterized by domains of affective disturbance, disturbed cognition, impulsivity and intense unstable relationships (Lieb et al., 2004). The Zanarini Rating Scale for Borderline Personality Disorder (ZAN‐BPD) was developed as an outcome measure for intervention research. It demonstrates good psychometric characteristics such as high levels of reliability, strong convergent validity with other measures, and sensitivity to detecting change in the severity of the symptoms of BPD (Zanarini, 2003; Zanarini et al., 2015). Hence, ZAN‐BPD has become widely used as a BPD specific outcome measure in recently conducted randomized clinical trials (RCTs; Black et al., 2014; Blum et al., 2008; Crawford et al., 2018; Hasler et al., 2014). However, there are no studies conducted independently of the designers of the measure that explore the factor structure of the ZAN‐BPD scale longitudinally over time in clinical samples of BPD patients.

Furthermore, there is a problem with construct validity affecting all BPD outcome measures based on DSM‐IV or V diagnostic criteria for BPD, including the ZAN‐BPD. None of them show consistent construct validity across studies, with the numbers of dimensions ranging from one to four (Becker et al., 2010; Clarkin et al., 1993; Clifton & Pilkonis, 2007; Leung & Leung, 2009; Sanislow, Morey, et al., 2002; Sanislow et al., 2000; Speranza et al., 2012). The nine‐item ZAN‐BPD was originally designed to evaluate affective, cognitive, impulsive and interpersonal symptoms, which are the four core areas of BPD psychopathology (Zanarini, 2003; Zanarini et al., 2015). However, the four‐factor structure was only reported cross‐sectionally in a study of a normal adolescent population screened with the MacLean Screening Instrument for BPD (Leung & Leung, 2009). In a clinical sample of adult BPD patients, the designers of the ZAN‐BPD found that the measure fitted a two‐factor model rather than the posited four‐factor structure, and the two‐factor structure was stable over two time points in a clinical sample (Zanarini et al., 2015).

There is a need to independently test the factor structure of the ZAN‐BPD in a large clinical sample of BPD patients measured longitudinally across more than two time points. An outcome measure suitable for intervention research as a primary outcome variable must show empirically that the meaning of questionnaire items and factor structure are both conceptually valid and stable across repeated measurement over time. Otherwise interventions might appear to be effective when they are not because change might be due to the unstable questionnaire construct over time. Using factor analysis, measurement equivalence or invariance (ME/I) indicates that the same constructs are being measured over time (Vandenberg & Lance, 2000).

To explore the dimensionality of the ZAN‐BPD as a measure, both exploratory factor analysis (EFA) and confirmatory factor analysis (CFA) have previously been used (Becker, Añez, et al., 2010; Becker et al., 2006; Johansen et al., 2004; Leung & Leung, 2009; Sanislow, Grilo, & McGlashan, 2000). However, recent methodology showed that both EFA and CFA have methodological limitations (Asparouhov & Muthén, 2009; Marsh, Morin, et al., 2014). EFA modelling cannot incorporate latent EFA factors into subsequent analyses. Moreover, it is difficult to test measure invariance across groups and/or times (Marsh, Morin, et al., 2014). When using CFA modelling, each item is strictly loaded on only one factor and all non‐target loadings are constrained to zero. The latest analytical approach, Exploratory Structural Equation Modelling **(**ESEM), integrates the best features of both EFA and CFA together. It applies EFA rigorously to specify more appropriately the underlying factor structure together with the advanced statistical methods typically associated with CFAs (Marsh, Morin, et al., 2014). ESEM allows cross item factor loadings that are coherent with the underlying theory and/or item contents so that items may cross load on different latent factors. ESEM reduces the bias in parameter estimates due to zero loading restriction that generally results in inflated CFA factor correlations. The latter might occur if items are not perfect factor indicators with some degree of irrelevant association with other constructs (Guay et al., 2015; Marsh, Morin, et al., 2014; Morin, Arens, & Marsh, 2016). Therefore, we used ESEM to explore the dimensionality of ZAN‐BPD in this study.

Using ZAN‐BPD item total scores to reflect the severity of BPD implies that there is one overarching general BPD factor on this measure (Zanarini, 2003; Zanarini et al., 2015). Bi‐factor models have statistical advantages over the traditional second order factor analytical model (Chen et al., 2006; Morin, Arens, & Marsh, 2016; Morin, Arens, Tran, & Caci, 2016). In bi‐factor models all items are simultaneously loaded on the overarching global factor with specific factors representing each of the a priori sub‐factors of the measure. We will explore if the ZAN‐BPD has an overarching general factor by means of bi‐factor modelling.

In summary, ESEM was performed to explore the construct validity of the ZAN‐BPD over time for BPD patients. The conceptual consistency of the ZAN‐BPD was examined by means of the ME/I procedure.

## 2 METHOD

### 2.1 Samples and ZAN‐BPD

Participants were 276 BPD patients (mean [sd] age = 36.1 [11] years, 208 [75.6%] female, 246 [89.13%] white, 200 [72.46%] unemployed). They were drawn from a multicentre, double‐blinded, two‐arm RCT comparing lamotrigine treatment effects over placebo with the ZAN‐BPD as the primary outcome measure (Crawford et al., 2018). Each participant met DSM‐IV criteria for borderline personality disorder, as assessed by the Structured Clinical Interview for DSM‐IV Axis II Personality Disorders (First et al., 1997). Further patient demographic and clinical information may be found in the trial report (Crawford et al., 2018). Patients' outcomes were evaluated at baseline and follow up at 12, 24 and 52 weeks after the randomization. Three participants withdrew shortly after randomisation and did not complete their baseline assessments so only 273 patients' data were included in the analysis.

The severity of BPD was evaluated by clinicians using the interview version of the ZAN‐BPD which has the following nine items for the four categories of BPD symptoms: affective symptoms (chronic angry/frequent angry acts; affective instability; chronic emptiness); cognitive symptoms (stress‐related paranoia/dissociation; serious identity disturbance); impulsivity symptoms (self‐destructive efforts; other impulsivity) and interpersonal symptoms (frantic efforts to avoid abandonment; stormy relationships). Each item was rated in order of severity with 0 = no, 1 = mild, 2 = moderate, 3 = serious and 4 = severe symptoms. The item total score, ranging from 0 to 36, indicated the level of symptoms and behavioural problems experienced by BPD patients. Comparisons of the ZAN‐BPD total score were made between arms at each follow up in the trial. At week 52 the mean (SD) totals were 11.3 (6.6) and 11.5 (7.7) for treatment arm and control arm respectively. There were no statistically significant differences between the two treatment arms on the ZAN‐BPD nor on any secondary outcome measure in this trial.

### 2.2 Statistics

The factor structure of the ZAN‐BPD was explored using ESEM (Marsh, Morin, et al., 2014). CFA was firstly conducted to replicate the posited ZAN‐BPD factor structure but failed (Supplementary Appendix A). We tested separately one to five first order factors and also bi‐factor models with one to four domain specific factors for data measured at each follow up time point. All aforementioned factor structures were further tested using all data measured at each time point stored in a wide format. Alike items factor loading parameters were set equal and unequal across all measurement time points. Ordinal item scores were analysed with the diagonally weighted least squares estimator using Delta parameterization and oblique rotation. Missing values were automatically accounted for using the full‐information maximum likelihood approach built into Mplus (Enders & Bandalos, 2001; Graham, 2003). Measurement invariance across all the follow‐up time points for the best fitted factor structure was tested using ESEM by sequentially testing the configural invariance model and scalar invariance model fittings (Fried et al., 2016; Muthén & Muthén, 2017; Vandenberg & Lance, 2000). The metric model is not allowed for ordinal items when ESEM is used (Muthén & Muthén, 2017). The configural invariance model tests whether the factor structure was the same on each occasion, meaning that the pattern of factor loadings on the indicators was the same across measurement waves. The scalar invariance model further set equal factor loadings for like items and equal threshold value of like items' regression on the latent variable(s) across measurement time points. All ESEM models were performed using software Mplus 8 (Muthén & Muthén, 2017).

Due to the sensitivity of the chi‐square (*χ* ^2^) test to large sample sizes and non‐normal data (Wen et al., 2004), the criteria for justifying good model fitting in this study are: both comparative fit index (CFI) and the non‐normed fit index (NNFI) > 0.95, Root Mean Square Error of Approximation (RMSEA) < 0.05 (Kline, 2015). Since the statistical data can only examine model fit and not the clinical relevance of factors, the factor loading estimates and item‐factor mapping pattern were additionally examined by two experienced psychiatrists (RM, MC). Model comparisons were generally evaluated by reference to the *χ* ^2^ change test using the Mplus DIFFTEST function to conduct *χ* ^2^ difference tests, as the WLSMV estimator was used to analyse ordinal items scores (Muthén & Muthén, 2017). The *χ* ^2^ change tests are influenced by sample size and data non‐normality (Cheung & Rensvold, 2002; Marsh, Muthén, et al., 2009; Vandenberg & Lance, 2000). Therefore the CFI change (drop ≥ 0.01) was used to compare model improvement, because CFI change is independent of both model complexity and sample size nor correlated with the overall fit measurements (Cheung & Rensvold, 2002; Vandenberg & Lance, 2000).

The data presented here is a secondary data analysis of data from a RCT (Crawford et al., 2018) that was powered to detect a minimum clinically important difference between the drug lamotrigine and placebo. We utilised all the data available from this RCT rather than carrying out a formal power calculation for the purposes of exploring the construct validity of the ZAN‐BPD over time.

## 3 RESULTS

### 3.1 Factor structure of ZAN‐BPD

When evaluating results of models with various latent factors in this study, we examined both the loading pattern consistency across models and mode fitting information for each model. The results of loading pattern and fitting comparison were summarised and presented in Supplementary table A2‐2 in appendix B. As to model fittings, the four‐factor and bi‐3 factor solutions did not converge at baseline so they were excluded from further consideration. The two‐factor and bi‐1 factor solutions had a RMSEA >0.05 at all four time points, a CFI <0.95 in both overall model and a NNFI <0.90 on the overall configural MI model. In contrast both the three‐factor and bi‐2 models had a much better fit to the data meeting all preset criteria at all time points and in both overall models except a CFI of 0.946 in the overall configural model just below the requirement for a CFI >0.95. This latter requirement was met in the overall loading MI model with a CFI of 0.962.

Examination of the items of the ZAN‐BPD across time for the 3‐factor and the bi‐2 factor solutions revealed that the bi‐2 factor solution had a much more stable structure across all time points and in the overall models. The bi‐2 factor seemed clinically meaningful. Clinically patients with BPD sometimes differ with respect to whether they externalise or internalise their distress (Factor 1). Some BPD patients display a lot of self‐harm associated with emptiness or low mood while others find it difficult to cope with interpersonal relationships and rarely self‐harm. Therefore, the bi‐2 factor structure is the best fitted and meaningful model. The model fitting information of the bi‐2 factor structure is presented in Table 1; all items loading estimates from the model with alike item loading estimates set equal across measurement times are presented in Table 2. All other ESEM modelling results are presented in Supplementary Appendix B.

**TABLE 1:** Model fitting information of bi‐2 factor structure for all analytical datasets

Data	*χ*^2^ (df), *p* =	RMSEA	CFI	NNFI	*N*
Baseline	12.202 (12), 0.4296	0.008	1.000	0.999	273
12 weeks	13.192 (12), 0.3552	0.021	0.9999	0.996	215
24 weeks	11.432 (12), 0.4923	0.000	1.000	1.002	196
52 weeks	10.405 (12), 0.5805	0.000	1.000	1.005	195
All data unconstrained	653.026 (480), 0.0001	0.036	0.946	0.930	273
All data fully constrained	656.694 (534), 0.0002	0.029	0.962	0.955	273

**TABLE 2:** Item factor loadings for Zanarini Rating Scale for Borderline Personality Disorder (bi‐2 factor model for overall data)

Item	G Factor	Factor 1	Factor 2
Chronic anger/frequent angry acts	**0.556 (*p* < 0.001)**	**−0.329 (*p* < 0.001)**	0.009 (*p* = 0.742)
Affective instability	**0.614 (*p* < 0.001)**	−0.012 (*p* = 0.828)	0.019 (*p* = 0.584)
Chronic emptiness	**0.393 (*p* < 0.001)**	**0.286 (*p* < 0.001)**	**−0.238 (*p* < 0.001)**
Stress‐related paranoia/dissociation	**0.588 (*p* < 0.001)**	**0.289 (*p* < 0.001)**	**0.093 (p = 0.039)**
Serious identity disturbance	**0.596 (*p* < 0.001)**	**0.355 (*p* < 0.001)**	−0.019 (*p* = 0.219)
Frantic efforts to avoid abandonment	**0.576 (*p* < 0.001)**	0.027 (*p* = 0.378)	**0.259 (*p* < 0.001)**
Self‐destructive efforts	**0.269 (*p* < 0.001)**	−0.012 (*p* = 0.089)	**−0.388 (*p* < 0.001)**
Other impulsivity	**0.563 (*p* < 0.001)**	**−0.482 (*p* < 0.001)**	−0.010 (*p* = 0.549)
Stormy relationships	**0.670 (*p* < 0.001)**	−0.092 (*p* = 0.250)	**0.296 (*p* < 0.001)**

**FIGURE 1:**
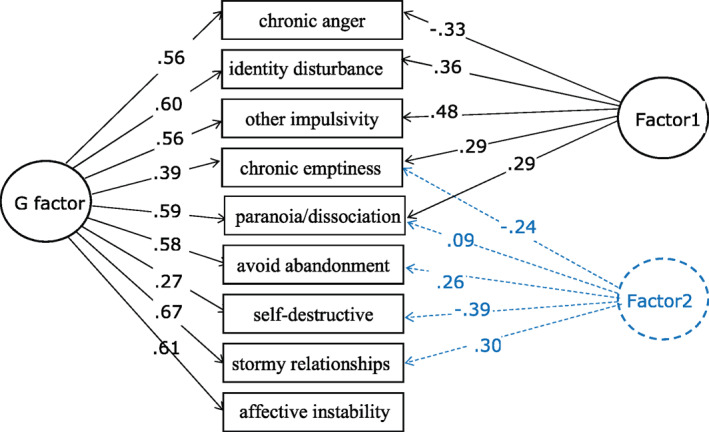
The schematic plot of bi‐2 factor model with significant item loadings as shown in Table 2

Table 2 shows there is evidence for a general BPD factor on the ZAN‐BPD and all items statistical significantly load on to this general factor. Furthermore, eight of the nine ZAN‐BPD items load more strongly on the general factor than Factors 1 and 2 (Figure 1). Factor 1 may be interpreted as a factor contrasting externalising distress through anger (chronic anger/frequent angry acts, other impulsivity) with internalising distress (chronic emptiness, stress‐related paranoia/dissociation and serious identity disturbance). Factor 2 may be interpreted as a factor contrasting depression and self‐destruction (chronic emptiness, self‐destructive acts) with interpersonal anxiety and conflict (frantic efforts to avoid abandonment, stormy relationships, stress‐related paranoia and dissociation).

### 3.2 The construct consistency across time

The modelling fittings of the ME/I test across measurement time are presented in Table 3. The configural invariance model (a) with default setting did not fit the data very well if strict criteria (0.90 < both CFI and NNFI <0.95) are applied. However, the modelling fitting was improved (Configural [b]) by correlating item 7 repeatedly measured at each time, which was guided by examining the modification index information in the configural (a) model (Vandenberg & Lance, 2000). The threshold invariance model (a) did not fit the data well (both CFI and NNFI <0.8) by its default setting. After checking the configural (b) model parameter estimates, 25 (17%) out of 144 threshold parameters were freely estimated and the model (threshold (b) fitting improved a lot when it was compared with the threshold (a) model. The CFI drop from the configural (b) to the threshold (b) model is ΔCFI = 0.010 so an invariant threshold model was retained. Nevertheless, the threshold (b) model is an acceptable and justifiable partial invariant threshold model (Vandenberg & Lance, 2000). Therefore, the ME/I test results showed that the bi‐2 factor structure is conceptually stable across measurement time points.

**TABLE 3:** Model fitting information for measurement equivalence or invariance across time (*n* = 273)

Variance model	*χ*^2^ (df), *p* =	RMSEA	CFI	NNFI	Δ*χ*2 (df), *p* =	ΔCFI
Configural a	653.026 (480),0.000	0.036	0.946	0.930		
Configural b^a^	597.008 (476),0.000	0.031	0.963	0.950		0.017
Threshold a	1312.844 (638),0.000	0.062	0.791	0.794	695.272 (162),0.000	0.155
Threshold b^b^	765.887 (613),0.000	0.030	0.953	0.951	194.808 (137),0.001	0.010

^a^ Correlate item_7 measured at each time but not between baseline and 52 weeks.

^b^ Free 25 (17%) out of 144 item threshold estimates.

## 4 DISCUSSION

In this study, the originally posited four factors of the ZAN‐BPD (Zanarini, 2003) were not replicated in a British BPD patient group. The results showed that a bi‐2 factor was the best fitting model at all time points. The item loading was stable over time and was clinically valid. The model was also conceptually stable across four measurement time points over 52 weeks. There is a general factor on which all nine items of the ZAN‐BPD loaded at all time points, and eight of the nine items loaded more strongly on the general factor rather than the specific factors supporting the use of the total score as a valid measure of outcome for clinical trials of interventions or for prospective longitudinal data. There are two specific factors that may be clinically useful for investigation as predictors of outcome in future studies.

The results of this study do not at first appear to support other previous factor analysis studies. However, this is the first time a bi‐factor model has been tested in a population of BPD participants. Previous hierarchical factor studies of the ZAN‐BPD (James & Taylor, 2008; Lai et al., 2012; Speranza et al., 2012; Zanarini, 2003; Zanarini et al., 2015) or other measures of BPD psychopathology (Blais et al., 1997; Clarkin et al., 1993; Sanislow, Grilo, & McGlashan, 2000) have found two to four factor solutions. These differences might relate to differences in the populations studied, or to different methods of statistical analysis. Moreover, these factors are often correlated with each other (Conway et al., 2012). Studies using other statistical approaches such as latent class, latent trait or item response theory often find a single unitary category of BPD.

(Clifton & Pilkonis, 2007; Conway et al., 2012; Feske et al., 2007; Fossati et al., 1999; Smits et al., 2017; Trull et al., 2011). Our current findings are therefore consistent with there being a single unitary general category of BPD symptoms.

Furthermore we confirm and extend a previous study by the designers of the measure over two time points in a United States adult clinical sample of BPD patients (Zanarini et al., 2015) by showing independently in a British clinical sample that the factor structure and item response of the ZAN‐BPD is generally stable over four time points. ZAN‐BPD was already shown to have good inter‐and intra‐rater reliabilities (Zanarini, 2003; Zanarini et al., 2015), while previous RCTs suggest that the measure is sensitive to change in BPD patients (Black et al., 2014). For rating scales to be used as a primary outcome in RCTs, medicine regulatory bodies require independent assessment to address the extent to which a rating scale measures what it is supposed to measure, inter‐ and intra‐rater reliability and responsiveness for detecting changes in the severity of disease (EMA, 1998). This study provides additional independent evidence of what ZAN‐BPD conceptually measures. Therefore, ZAN‐BPD is a good outcome measure for RCTs with longitudinal designs because it meets regulatory requirements.

The two factors that we found in addition to the general factor are of potential clinical significance and might provide additional information to investigators as predictors of outcome. They should not be used as outcome measures because eight of the nine ZAN‐BPD items load more strongly on the general factor than the specific factors. Chronic anger or frequent angry acts and other impulsivity, can be viewed as externalising distress, and these items are negatively scored on Factor 1. Chronic emptiness, stress related paranoia or dissociation and serious identity disturbance, are all forms of internalised distress that are positively scored on Factor 1. Personality dysfunction including BPD may be considered in terms of externalising or outward directed distress such as impulsive behaviour and internalised distress such as affective and cognitive symptoms (Eaton et al., 2011; Lai et al., 2012; Whewell et al., 2000). Factor 2 contrasts patients with BPD who rarely carry out suicidal acts and display a lot of anxiety and stress‐related symptoms while others show chronic emptiness and carry out frequent suicidal acts (Whewell et al., 2000). Chronic emptiness and self‐destructive acts, thought to be clinically associated with increased risk of suicide attempts as well as self‐harm (Blasco‐Fontecilla et al., 2013; Klonsky, 2008; Miller et al., 2020), were negatively scored on Factor 2. Frantic efforts to avoid abandonment, stormy relationships, and stress related paranoia or dissociation have been related to non‐suicidal self‐injury (Brickman et al., 2014) and are positively weighted on Factor 2. Affective instability is a core symptom of BPD so reassuringly it loaded strongly on the general factor (American Psychiatric Association, 2013). However, it did not load significantly on Factor 1 or Factor 2 because it may take externalising or internalising forms and is a feature of both anxiety and stress‐related symptoms or suicidal acts and emptiness. Factor 1 may be worth consideration as a predictor of outcome in studies concerned with expression of BPD distress while Factor 2 is worth further exploration in studies with BPD patients exploring suicidality and non‐suicidal self‐injury. However, further independent research is needed to test both factors 1 and 2, including whether these items are stable traits over time or more state dependent dimensions of BPD.

Strengths of this study included a well‐characterised multicentre clinical sample diagnosed using a standardised psychiatric interview and DSM‐V criteria for BPD. The participants were from a RCT with high rates of follow up over four time points across 52 weeks. We explored longitudinal change using ZAN‐BPD, a measure designed to assess outcome with sensitivity to change in RCTs. We employed the latest factor analytical approach ESEM to explore the dimensionality of ZAN‐BPD and its conceptual stability across four time points over 52 weeks.

The limitations of this study include a smaller sample size at baseline compared to some other cross‐sectional ZAN‐BPD factor analysis studies for example, Clifton and Pilkonis (2007), Leung and Leung (2009), Sanislow, Morey, et al. (2002). However, the sample size is larger than some other previous factor analysis studies for example, Becker, Añez, et al. (2010), Speranza et al. (2012), Zanarini et al. (2015), and has more follow up time points and data than previous studies. The sample size calculation was based on assessing treatment effects on the ZAN‐BPD as a primary outcome measure in the RCT rather than the factor structure of the ZAN‐BPD over time. Hence the sample size might not be sufficient to test every parameter in the targeting models (see Supplementary Appendix C). Other models might show improved fit to the data with larger sample sizes so further independent replication of these results would be welcome.

A concern is that bifactor modelling may overfit the data compared to higher order models, especially if there is over‐reliance on goodness of fit indices and insufficient attention to their interpretability (Hyland et al., 2020). Further ancillary analyses have been proposed to evaluate dimensionality (e.g., presenting epidemiological cut‐off values, internal versus external cross‐validation, and average relative parameter bias) and reliability of the models (Omega, Omega Hierarchical, Omega Hierarchical Subscale, and PRV) to statistically assess whether the bi‐2 factor model or a higher order factor is the most appropriate model (Rodriguez et al., 2016a, 2016b). However, these model evaluating indices were not suitable to evaluate the bifactor model in our study because our model items were ordinal and the two group factors were correlated. Importantly the bi‐2 factor model showed a more stable item structure over multiple time points than the 3‐factor model, and was clinically interpretable. However, reliance on expert clinical interpretation to validate the specific factors is a limitation without additional concurrently applied validated measures.

Another limitation is that the sample consisted of those who agreed to take part in an RCT involving treatment with lamotrigine and a placebo (Crawford et al., 2018). However, the inclusion criteria were broad in this pragmatic trial and one of the main uses of the ZAN‐BPD is as an outcome measure in clinical trials. The majority of participants were white British adults and all were in contact with mental health services so the results may not generalise to adolescents, community samples, non‐clinical samples or people with a different ethnic and cultural background.

In summary, in adult BPD patients ZAN‐BPD measure has a bi‐2 factor structure with a general factor to which all nine scale items contribute and two factors that may contribute additional information on externalising or internalising distress, or depression and self‐destruction versus interpersonal anxiety and conflict. Its dimensional structure is conceptually stable across time. Therefore, ZAN‐BPD is a conceptually valid scale to measure total BPD severity that is suitable for longitudinal and intervention studies. The two sub‐factors may be utilised as predictors of outcome but are not outcome measures in their own right.

## CONFLICT OF INTERESTS

The authors declared no potential conflict of interest with respect to the research, authorship, and/or publication of this article.

## Supporting information

Supplementary Material

## Data Availability

The data that support the findings of this study are available on request from the corresponding author. The data are not publicly available due to ethical restrictions.
