# Supplementary material for: The factor structure of the Zanarini Rating Scale for Borderline Personality Disorder: Exploratory Structural Equation Modelling and measurement invariance over time
Source: Int J Methods Psychiatr Res. 2021 May 12;30(3):e1874. doi: 10.1002/mpr.1874 (PMC8412222; doi:10.1002/mpr.1874)
Supplement: Supplementary file 1 — Supplementary Material [file MPR-30-e1874-s001.docx]

**Appendix A**

**Results of original four-factor structure model fitting for ZAN-BPD**

Table A1: CFA model fittings information of four-factor CFA using all time points and overall data.

| Data | N | χ2 (df), p= | | RMSEA | | CFI | NNFI | | SRMR |  |
| --- | --- | --- | --- | --- | --- | --- | --- | --- | --- | --- |
| Baseline | 273 | 92.499(21), p < 0.0001 | | 0.112 | | 0.904 | 0.835 | | 0.054 |  |
| 12 week | 215 | 69.748(21), p < 0.0001 | | 0.104 | | 0.942 | 0.900 | | 0.050 |  |
| 24 week | 196 | 84.322(21), p < 0.0001 | | 0.124 | | 0.931 | 0.882 | | 0.055 |  |
| 52 week | 195 | 90.068(21), p < 0.0001 | | 0.130 | | 0.932 | 0.884 | | 0.051 |  |
| Overall* configural MI | 273 | 901.190(474), p < 0.0001 | | 0.057 | | 0.868 | 0.824 | | 0.080 |  |
| Overall loading MI | 273 | 882.420(489), p < 0.0001 | | 0.054 | | 0.878 | 0.843 | | 0.082 |  |
| * all repeated data were analysed simultaneously; MI: measurement invariance test. Same note for all tables in this document.  Table A1-1: CFA Item four-factor loading for ZAN-BPD (baseline) | | | | | | | | | | |
| Item | | | Factor 1 | | Factor 2 | | | Factor 3 | | Factor 4 |
| 1. chronic angry/frequent angry acts | | | 0.615(p<0.001) | |  | | |  | |  |
| 2. affective instability | | | 0.665(p<0.001) | |  | | |  | |  |
| 3. chronic emptiness | | | 0.294(p<0.001) | |  | | |  | |  |
| 4. stress-related paranoia/ dissociation | | |  | | 0.720(p<0.001) | | |  | |  |
| 5. serious identity disturbance | | |  | | 0.649(p<0.001) | | |  | |  |
| 6. frantic efforts to avoid abandonment | | |  | |  | | | 0.584(p<0.001) | |  |
| 7. self-destructive efforts | | |  | |  | | |  | | 0.190(p=0.053) |
| 8. other impulsivity | | |  | |  | | |  | | 0.405(p=0.021) |
| 9. stormy relationships | | |  | |  | | | 0.759(p<0.001) | |  |

| Table A1-2: CFA Item four-factor loading for ZAN-BPD (12 week) | | | | |
| --- | --- | --- | --- | --- |
| Item | Factor 1 | Factor 2 | Factor 3 | Factor 4 |
| 1. chronic angry/frequent angry acts | 0.630(p<0.001) |  |  |  |
| 2. affective instability | 0.686(p<0.001) |  |  |  |
| 3. chronic emptiness | 0.555(p<0.001) |  |  |  |
| 4. stress-related paranoia/ dissociation |  | 0.733(p<0.001) |  |  |
| 5. serious identity disturbance |  | 0.736(p<0.001) |  |  |
| 6. frantic efforts to avoid abandonment |  |  | 0.534(p<0.001) |  |
| 7. self-destructive efforts |  |  |  | 0.497(p<0.001) |
| 8. other impulsivity |  |  |  | 0.615(p<0.001) |
| 9. stormy relationships |  |  | 0.827(p<0.001) |  |

| Table A1-3: CFA Item four-factor loading for ZAN-BPD (24 week) | | | | |
| --- | --- | --- | --- | --- |
| Item | Factor 1 | Factor 2 | Factor 3 | Factor 4 |
| 1. chronic angry/frequent angry acts | 0.648(p<0.001) |  |  |  |
| 2. affective instability | 0.745(p<0.001) |  |  |  |
| 3. chronic emptiness | 0.597(p<0.001) |  |  |  |
| 4. stress-related paranoia/ dissociation |  | 0.818(p<0.001) |  |  |
| 5. serious identity disturbance |  | 0.771(p<0.001) |  |  |
| 6. frantic efforts to avoid abandonment |  |  | 0.672(p<0.001) |  |
| 7. self-destructive efforts |  |  |  | 0.386(p<0.001) |
| 8. other impulsivity |  |  |  | 0.566(p<0.001) |
| 9. stormy relationships |  |  | 0.715(p<0.001) |  |

| Table A1-4: CFA Item four-factor loading for ZAN-BPD (52 week) | | | | |
| --- | --- | --- | --- | --- |
| Item | Factor 1 | Factor 2 | Factor 3 | Factor 4 |
| 1. chronic angry/frequent angry acts | 0.744(p<0.001) |  |  |  |
| 2. affective instability | 0.782(p<0.001) |  |  |  |
| 3. chronic emptiness | 0.620(p<0.001) |  |  |  |
| 4. stress-related paranoia/ dissociation |  | 0.639(p<0.001) |  |  |
| 5. serious identity disturbance |  | 0.762(p<0.001) |  |  |
| 6. frantic efforts to avoid abandonment |  |  | 0.702(p<0.001) |  |
| 7. self-destructive efforts |  |  |  | 0.533(p<0.001) |
| 8. other impulsivity |  |  |  | 0.671(p<0.001) |
| 9. stormy relationships |  |  | 0.800(p<0.001) |  |

**Note: None of the CFA four-factor models fitted the data well.**

**Appendix B**

**Results of factor structure for ZAN-BPD by ESEM analysis**

| Table A2-1:Model fitting indices for one-, two-, three- and four-factor modelling and bi-factor modelling | | | | | | | |  | |
| --- | --- | --- | --- | --- | --- | --- | --- | --- | --- |
| Data | Model | χ2 (df), p= | RMSEA | CFI | NNFI | Δχ2 (df), p= | Model compare | | |
| baseline | 1.ESEM 1 factor | 112.387(27), p < 0.0001 | 0.108 | 0.885 | 0.847 | 60.168(8), p < 0.0001 | | | 1 vs. 2 |
| (n = 273) | 2.ESEM 2 factor | 36.641(19), p = 0.0088 | 0.058 | 0.976 | 0.955 | 21.788(7), p = 0.0028 | | | 2 vs. 3 |
|  | 3.ESEM 3 factor | 12.202(12), p = 0.4296 | 0.008 | 1.000 | 0.999 |  | | |  |
|  | 4.ESEM 4 factor | Not convergent |  |  |  |  | | |  |
|  | 5.CFA 4 factor | 92.499(21), p < 0.0001 | 0.112 | 0.904 | 0.835 |  | | |  |
|  | 6.ESEM bi-factor 1 | 36.641(19), p = 0.0088 | 0.058 | 0.976 | 0.955 |  | | |  |
|  | 7.ESEM bi-factor 2 | 12.202(12), p = 0.4296 | 0.008 | 1.000 | 0.999 |  | | |  |
|  | 8.ESEM bi-factor 3 | Not convergent |  |  |  |  | | |  |
|  | 9.ESEM bi-factor 4 | Not convergent |  |  |  |  | | |  |
|  |  |  |  |  |  |  | | |  |
| 12 week | 1.ESEM 1 factor | 87.980(27), p < 0.0001 | 0.102 | 0.927 | 0.903 | 40.844(8), p < 0.0001 | | | 1 vs. 2 |
| (n = 215) | 2.ESEM 2 factor | 40.281(19), p = 0.0030 | 0.072 | 0.974 | 0.952 | 24.036(7), p = 0.0011 | | | 2 vs. 3 |
|  | 3.ESEM 3 factor | 13.192(12), p = 0.3552 | 0.021 | 0.999 | 0.996 | 9.897(6), p = 0.1291 | | | 3 vs. 4 |
|  | 4.ESEM 4 factor | 3.704(6), p = 0.7166 | 0.000 | 1.000 | 1.017 |  | | |  |
|  | 5.ESEM bi-factor 1 | 40.281(19), p = 0.0030 | 0.072 | 0.974 | 0.952 |  | | |  |
|  | 6.ESEM bi-factor 2 | 13.192(12), p = 0.3552 | 0.021 | 0.999 | 0.996 |  | | |  |
|  | 7.ESEM bi-factor 3 | 3.704(6), p = 0.7166 | 0.000 | 1.000 | 1.017 |  | | |  |
|  | 8.ESEM bi-factor 4 | Not convergent |  |  |  |  | | |  |
|  |  |  |  |  |  |  | | |  |
| 24 week | 1.ESEM 1 factor | 124.945(27), p < 0.0001 | 0.136 | 0.893 | 0.858 | 74.536(8), p < 0.0001 | | | 1 vs. 2 |
| (n = 196) | 2.ESEM 2 factor | 30.326(19), p = 0.0478 | 0.055 | 0.988 | 0.977 | 17.530(7), p = 0.0143 | | | 2 vs. 3 |
|  | 3.ESEM 3 factor | 11.432(12), p = 0.4923 | 0.000 | 1.000 | 1.002 | 9.418(6), p = 0.1514 | | | 3 vs. 4 |
|  | 4.ESEM 4 factor | 1.021(6), p = 0.9848 | 0.000 | 1.000 | 1.032 | 0.897(5), p = 0.9704 | | | 4 vs. 8 |
|  | 5.ESEM bi-factor 1 | 30.326(19), p = 0.0478 | 0.055 | 0.988 | 0.977 |  | | |  |
|  | 6.ESEM bi-factor 2 | 11.432(12), p = 0.4923 | 0.000 | 1.000 | 1.002 |  | | |  |
|  | 7.ESEM bi-factor 3 | 1.021(6), p = 0.9848 | 0.000 | 1.000 | 1.032 |  | | |  |
|  | 8.ESEM bi-factor 4 | 0.064(1), p = 0.8007 | 0.000 | 1.000 | 1.037 |  | | |  |
|  |  |  |  |  |  |  | | |  |
| 52 week | 1.ESEM 1 factor | 104.707(27), p < 0.0001 | 0.121 | 0.924 | 0.899 | 50.160(8), p < 0.0001 | | | 1 vs. 2 |
| (n = 195) | 2.ESEM 2 factor | 47.780(19), p = 0.0003 | 0.088 | 0.972 | 0.947 | 31.799(7), p < 0.0001 | | | 2 vs. 3 |
|  | 3.ESEM 3 factor | 10.405(12), p = 0.5805 | 0.000 | 1.000 | 1.005 | 7.813(6), p = 0.2521 | | | 3 vs. 4 |
|  | 4.ESEM 4 factor | 2.253(6), p = 0.8950 | 0.000 | 1.000 | 1.022 | 1.222(5), p = 0.9427 | | | 4 vs. 8 |
|  | 5.ESEM bi-factor 1 | 47.780(19), p = 0.0003 | 0.088 | 0.972 | 0.947 |  | | |  |
|  | 6.ESEM bi-factor 2 | 10.405(12), p = 0.5805 | 0.000 | 1.000 | 1.005 |  | | |  |
|  | 7.ESEM bi-factor 3 | 2.253(6), p = 0.8950 | 0.000 | 1.000 | 1.022 |  | | |  |
|  | 8.ESEM bi-factor 4 | 1.466(1), p = 0.2260 | 0.049 | 1.000 | 0.984 |  | | |  |
|  |  |  |  |  |  |  | | |  |
| overall | 1.ESEM 1 factor | 1278.340(588), p < 0.0001 | 0.066 | 0.786 | 0.771 |  | | |  |
| configual MI | 2.ESEM 2 factor | 895.487(538), p < 0.0001 | 0.049 | 0.889 | 0.870 |  | | |  |
| (n = 273) | 3.ESEM 3 factor | 653.026(480), p < 0.0001 | 0.036 | 0.946 | 0.930 |  | | |  |
|  | 4.ESEM 4 factor | 506.055(414), p = 0.0013 | 0.029 | 0.972 | 0.957 |  | | |  |
|  | 5.ESEM bi-factor 1 | 895.487(538), p < 0.0001 | 0.049 | 0.889 | 0.870 |  | | |  |
|  | 6.ESEM bi-factor 2 | 653.026(480), p < 0.0001 | 0.036 | 0.946 | 0.930 |  | | |  |
|  | 7.ESEM bi-factor 3 | 506.055(414), p = 0.0013 | 0.029 | 0.972 | 0.957 |  | | |  |
|  | 8.ESEM bi-factor 4 | 386.458(340), p = 0.0417 | 0.022 | 0.986 | 0.973 |  | | |  |
|  |  |  |  |  |  |  | | |  |
| overall | 1.ESEM 1 factor | 1241.911(612), p < 0.0001 | 0.061 | 0.805 | 0.799 |  | | |  |
| loading MI | 2.ESEM 2 factor | 858.167(580), p < 0.0001 | 0.042 | 0.914 | 0.907 |  | | |  |
| (n = 273) | 3.ESEM 3 factor | 656.694(534), p = 0.0002 | 0.029 | 0.962 | 0.955 |  | | |  |
|  | 4.ESEM 4 factor | 532.860(474), p = 0.0315 | 0.021 | 0.982 | 0.976 |  | | |  |
|  | 5.ESEM bi-factor 1 | 858.167(580), p < 0.0001 | 0.042 | 0.914 | 0.907 |  | | |  |
|  | 6.ESEM bi-factor 2 | 656.694(534), p = 0.0002 | 0.029 | 0.962 | 0.955 |  | | |  |
|  | 7.ESEM bi-factor 3 | 532.860(474), p = 0.0315 | 0.021 | 0.982 | 0.976 |  | | |  |
|  | 8.ESEM bi-factor 4 | 423.525(400), p = 0.2006 | 0.015 | 0.993 | 0.989 |  | | |  |

| Table A3-1: Item factor loading for ZAN-BPD (one factor at each time point) | | | | | | | | | | |  |
| --- | --- | --- | --- | --- | --- | --- | --- | --- | --- | --- | --- |
| Item | | Factor 1 at baseline | | Factor 1 at 12 week | | | Factor 1 at 24 week | | Factor 1 at 52 week | |  |
| 1. chronic angry/frequent angry acts | | 0.598(p<0.001) | | 0.632(p<0.001) | | | 0.652(p<0.001) | | 0.730(p<0.001) | |  |
| 2. affective instability | | 0.646(p<0.001) | | 0.690(p<0.001) | | | 0.744(p<0.001) | | 0.771(p<0.001) | |  |
| 3. chronic emptiness | | 0.294(p<0.001) | | 0.557(p<0.001) | | | 0.602(p<0.001) | | 0.612(p<0.001) | |  |
| 4. stress-related paranoia/ dissociation | | 0.617(p<0.001) | | 0.664(p<0.001) | | | 0.718(p<0.001) | | 0.609(p<0.001) | |  |
| 5. serious identity disturbance | | 0.571(p<0.001) | | 0.665(p<0.001) | | | 0.680(p<0.001) | | 0.722(p<0.001) | |  |
| 6. frantic efforts to avoid abandonment | | 0.545(p<0.001) | | 0.475(p<0.001) | | | 0.587(p<0.001) | | 0.593(p<0.001) | |  |
| 7. self-destructive efforts | | 0.250(p<0.001) | | 0.473(p<0.001) | | | 0.393(p<0.001) | | 0.538(p<0.001) | |  |
| 8. other impulsivity | | 0.531(p<0.001) | | 0.572(p<0.001) | | | 0.573(p<0.001) | | 0.676(p<0.001) | |  |
| 9. stormy relationships | | 0.682(p<0.001) | | 0.672(p<0.001) | | | 0.620(p<0.001) | | 0.655(p<0.001) | |  |
| Table A3-2: Item factor loading for ZAN-BPD (one factor for overall data) | | | | | | | | | | | |
| Item | Factor 1  at baseline | | Factor 1  at 12 week | | | Factor 1  at 24 week | | Factor 1  at 52 week | | Factor1 loading MI | |
| 1. chronic angry/frequent angry acts | 0.623(p<0.001) | | 0.614(p<0.001) | | | 0.596(p<0.001) | | 0.723(p<0.001) | | 0.570(p<0.001) | |
| 2. affective instability | 0.633(p<0.001) | | 0.673(p<0.001) | | | 0.679(p<0.001) | | 0.719(p<0.001) | | 0.605(p<0.001) | |
| 3. chronic emptiness | 0.296(p<0.001) | | 0.532(p<0.001) | | | 0.615(p<0.001) | | 0.607(p<0.001) | | 0.472(p<0.001) | |
| 4. stress-related paranoia/ dissociation | 0.487(p<0.001) | | 0.611(p<0.001) | | | 0.715(p<0.001) | | 0.647(p<0.001) | | 0.558(p<0.001) | |
| 5. serious identity disturbance | 0.503(p<0.001) | | 0.643(p<0.001) | | | 0.784(p<0.001) | | 0.726(p<0.001) | | 0.601(p<0.001) | |
| 6. frantic efforts to avoid abandonment | 0.553(p<0.001) | | 0.508(p<0.001) | | | 0.531(p<0.001) | | 0.625(p<0.001) | | 0.493(p<0.001) | |
| 7. self-destructive efforts | 0.369(p<0.001) | | 0.470(p<0.001) | | | 0.428(p<0.001) | | 0.502(p<0.001) | | 0.399(p<0.001) | |
| 8. other impulsivity | 0.699(p<0.001) | | 0.666(p<0.001) | | | 0.615(p<0.001) | | 0.707(p<0.001) | | 0.596(p<0.001) | |
| 9. stormy relationships | 0.619(p<0.001) | | 0.692(p<0.001) | | | 0.602(p<0.001) | | 0.658(p<0.001) | | 0.574(p<0.001) | |
| Table A4-1: Item factor loading for ZAN-BPD (two factors at baseline and 12 weeks) | | | | | | | | | | |  |
| Item | | Baseline | | | | | 12 week | | | |  |
|  |  | Factor 1 | | | Factor 2 | | Factor 1 | | Factor 2 | |  |
| 1. chronic angry/frequent angry acts | | 0.004(p=0.525) | | 0.685(p<0.001) | | | 0.000(p=0.981) | | 0.722(p<0.001) | |  |
| 2. affective instability | | 0.425(p<0.001) | | 0.325(p<0.001) | | | 0.360(p=0.001) | | 0.420(p<0.001) | |  |
| 3. chronic emptiness | | 0.495(p<0.001) | | -0.151(p=0.137) | | | 0.669(p<0.001) | | -0.049(p=0.655) | |  |
| 4. stress-related paranoia/ dissociation | | 0.632(p<0.001) | | 0.106(p=0.274) | | | 0.733(p<0.001) | | 0.005(p=0.954) | |  |
| 5. serious identity disturbance | | 0.686(p<0.001) | | -0.001(p=0.866) | | | 0.752(p<0.001) | | 0.003(p=0.960) | |  |
| 6. frantic efforts to avoid abandonment | | 0.320(p=0.001) | | 0.311(p=0.001) | | | 0.219(p=0.054) | | 0.318(p=0.004) | |  |
| 7. self-destructive efforts | | 0.361(p<0.001) | | -0.067(p=0.503) | | | 0.309(p=0.012) | | 0.226(p=0.071) | |  |
| 8. other impulsivity | | -0.100(p=0.378) | | 0.710(p<0.001) | | | -0.049(p=0.724) | | 0.696(p<0.001) | |  |
| 9. stormy relationships | | 0.168(p=0.080) | | 0.636(p<0.001) | | | 0.122(p=0.343) | | 0.639(p<0.001) | |  |

| Table A4-2: Item factor loading for ZAN-BPD (two factors at 24 and 52 weeks) | | | | | |
| --- | --- | --- | --- | --- | --- |
| Item | 24 week | | | 52 week | |
|  | Factor 1 | | Factor 2 | Factor 1 | Factor 2 |
| 1. chronic angry/frequent angry acts | 0.017(p=0.657) | 0.782(p<0.001) | | 0.913(p<0.001) | -0.140(p=0.326) |
| 2. affective instability | 0.474(p<0.001) | 0.393(p<0.001) | | 0.680(p<0.001) | 0.154(p=0.177) |
| 3. chronic emptiness | 0.625(p<0.001) | 0.033(p=0.743) | | 0.004(p=0.960) | 0.704(p<0.001) |
| 4. stress-related paranoia/ dissociation | 0.759(p<0.001) | 0.005(p=0.539) | | 0.336(p=0.001) | 0.348(p=0.003) |
| 5. serious identity disturbance | 0.908(p<0.001) | -0.175(p=0.102) | | -0.002(p=0.962) | 0.887(p<0.001) |
| 6. frantic efforts to avoid abandonment | 0.401(p<0.001) | 0.277(p=0.011) | | 0.425(p<0.001) | 0.229(p=0.057) |
| 7. self-destructive efforts | 0.308(p=0.002) | 0.141(p=0.193) | | 0.241(p=0.061) | 0.374(p=0.005) |
| 8. other impulsivity | -0.080(p=0.383) | 0.768(p<0.001) | | 0.717(p<0.001) | 0.002(p=0.793) |
| 9. stormy relationships | 0.289(p=0.003) | 0.445(p<0.001) | | 0.539(p<0.001) | 0.174(p=0.114) |
| Table A4-3: Item factor loading for ZAN-BPD (two factors for overall data) | | | | | |
| Item | Baseline | | | 12 week | |
|  | Factor 1 | | Factor 2 | Factor 1 | Factor 2 |
| 1. chronic angry/frequent angry acts | 0.143(p=0.046) | 0.593(p<0.001) | | 0.002(p=0.834) | 0.658(p<0.001) |
| 2. affective instability | 0.539(p<0.001) | 0.247(p=0.002) | | 0.351(p<0.001) | 0.426(p<0.001) |
| 3. chronic emptiness | 0.556(p<0.001) | -0.182(p=0.018) | | 0.683(p<0.001) | -0.048(p=0.589) |
| 4. stress-related paranoia/ dissociation | 0.629(p<0.001) | -0.010(p=0.858) | | 0.632(p<0.001) | 0.087(p=0.366) |
| 5. serious identity disturbance | 0.624(p<0.001) | 0.003(p=0.904) | | 0.776(p<0.001) | -0.001(p=0.846) |
| 6. frantic efforts to avoid abandonment | 0.382(p<0.001) | 0.296(p<0.001) | | 0.250(p=0.004) | 0.333(p<0.001) |
| 7. self-destructive efforts | 0.384(p<0.001) | 0.076(p=0.349) | | 0.407(p<0.001) | 0.145(p=0.180) |
| 8. other impulsivity | -0.009(p=0.193) | 0.786(p<0.001) | | -0.028(p=0.765) | 0.744(p<0.001) |
| 9. stormy relationships | 0.338(p<0.001) | 0.414(p<0.001) | | 0.137(p=0.139) | 0.633(p<0.001) |

| Table A4-4: Item factor loading for ZAN-BPD (two factors for overall data) | | | | | | |
| --- | --- | --- | --- | --- | --- | --- |
| Item | 24 week | | 52 week | | Overall loading MI | |
|  | Factor 1 | Factor 2 | Factor 1 | Factor 2 | Factor 1 | Factor 2 |
| 1. chronic angry/frequent angry acts | 0.012(p=0.480) | 0.698(p<0.001) | 0.765(p<0.001) | 0.013(p=0.609) | 0.668(p<0.001) | 0.014(p=0.394) |
| 2. affective instability | 0.400(p<0.001) | 0.404(p<0.001) | 0.551(p<0.001) | 0.242(p=0.003) | 0.386(p<0.001) | 0.333(p<0.001) |
| 3. chronic emptiness | 0.676(p<0.001) | -0.005(p=0.94) | -0.073(p=0.46) | 0.752(p<0.001) | -0.092(p=0.10) | 0.614(p<0.001) |
| 4. stress-related paranoia/ dissociation | 0.670(p<0.001) | 0.121(p=0.140) | 0.246(p=0.011) | 0.480(p<0.001) | 0.088(p=0.103) | 0.548(p<0.001) |
| 5. serious identity disturbance | 0.857(p<0.001) | -0.005(p=0.90) | 0.010(p=0.861) | 0.805(p<0.001) | 0.001(p=0.830) | 0.676(p<0.001) |
| 6. frantic efforts to avoid abandonment | 0.227(p=0.024) | 0.417(p<0.001) | 0.426(p<0.001) | 0.264(p=0.029) | 0.368(p<0.001) | 0.222(p<0.001) |
| 7. self-destructive efforts | 0.398(p<0.001) | 0.085(p=0.426) | 0.008(p=0.928) | 0.566(p<0.001) | 0.092(p=0.177) | 0.369(p<0.001) |
| 8. other impulsivity | -0.139(p=0.10) | .877(p<0.001) | 0.914(p<0.001) | -0.168(p=0.10) | 0.828(p<0.001) | -0.117(p=0.01) |
| 9. stormy relationships | 0.161(p=0.092) | 0.573(p<0.001) | 0.533(p<0.001) | 0.191(p=0.104) | 0.532(p<0.001) | 0.158(p=0.002) |

| Table A5-1: Item factor loading for ZAN-BPD (bi-factor 1 at baseline and 12 weeks) | | | | | |
| --- | --- | --- | --- | --- | --- |
| Item | Baseline | | | 12 week | |
|  | Factor G | | Factor 1 | Factor G | Factor 1 |
| 1. chronic angry/frequent angry acts | 0.595(p<0.001) | -0.342(p<0.001) | | 0.634(p<0.001) | 0.346(p<0.001) |
| 2. affective instability | 0.643(p<0.001) | 0.059(p=0.492) | | 0.690(p<0.001) | 0.038(p=0.607) |
| 3. chronic emptiness | 0.291(p<0.001) | 0.335(p<0.001) | | 0.554(p<0.001) | -0.326(p<0.001) |
| 4. stress-related paranoia/ dissociation | 0.631(p<0.001) | 0.277(p=0.001) | | 0.658(p<0.001) | -0.329(p=0.001) |
| 5. serious identity disturbance | 0.584(p<0.001) | 0.359(p<0.001) | | 0.674(p<0.001) | -0.338(p<0.001) |
| 6. frantic efforts to avoid abandonment | 0.542(p<0.001) | 0.011(p=0.920) | | 0.475(p<0.001) | 0.053(p=0.571) |
| 7. self-destructive efforts | 0.250(p<0.001) | 0.222(p=0.015) | | 0.473(p<0.001) | -0.032(p=0.769) |
| 8. other impulsivity | 0.528(p<0.001) | -0.409(p<0.001) | | 0.568(p<0.001) | 0.355(p<0.001) |
| 9. stormy relationships | 0.693(p<0.001) | -0.232(p=0.007) | | 0.670(p<0.001) | 0.250(p=0.004) |

| Table A5-2: Item factor loading for ZAN-BPD (bi-factor 1 at 24 and 52 weeks) | | | | | | | | | | | |  |
| --- | --- | --- | --- | --- | --- | --- | --- | --- | --- | --- | --- | --- |
| Item | | 24 week | | | | | | 52 week | | | |  |
|  |  | Factor G | | | Factor 1 | | | Factor G | | Factor 1 | |  |
| 1. chronic angry/frequent angry acts | | 0.645(p<0.001) | | 0.457(p<0.001) | | | | 0.733(p<0.001) | | -0.404(p<0.001) | |  |
| 2. affective instability | | 0.751(p<0.001) | | 0.044(p=0.739) | | | | 0.765(p<0.001) | | -0.163(p=0.021) | |  |
| 3. chronic emptiness | | 0.599(p<0.001) | | -0.231(p=0.044) | | | | 0.601(p<0.001) | | 0.373(p<0.001) | |  |
| 4. stress-related paranoia/ dissociation | | 0.700(p<0.001) | | -0.301(p=0.015) | | | | 0.608(p<0.001) | | 0.064(p=0.498) | |  |
| 5. serious identity disturbance | | 0.691(p<0.001) | | -0.467(p<0.001) | | | | 0.750(p<0.001) | | 0.473(p<0.001) | |  |
| 6. frantic efforts to avoid abandonment | | 0.590(p<0.001) | | 0.004(p=0.982) | | | | 0.590(p<0.001) | | -0.031(p=0.737) | |  |
| 7. self-destructive efforts | | 0.395(p<0.001) | | -0.040(p=0.726) | | | | 0.541(p<0.001) | | 0.112(p=0.297) | |  |
| 8. other impulsivity | | 0.545(p<0.001) | | 0.487(p<0.001) | | | | 0.671(p<0.001) | | -0.258(p=0.001) | |  |
| 9. stormy relationships | | 0.622(p<0.001) | | 0.148(p=0.214) | | | | 0.650(p<0.001) | | -0.102(p=0.260) | |  |
| Table A5-3: Item factor loading for ZAN-BPD (bi-factor 1 at baseline and 12 weeks) | | | | | | | | | | | |  |
| Item | | Baseline | | | | | | 12 week | | | |  |
|  |  | Factor G | | | Factor 1 | | | Factor G | | Factor 1 | |  |
| 1. chronic angry/frequent angry acts | | 0.580(p<0.001) | | -0.317(p<0.001) | | | | 0.571(p<0.001) | | -0.328(p<0.001) | |  |
| 2. affective instability | | 0.667(p<0.001) | | 0.089(p=0.257) | | | | 0.681(p<0.001) | | -0.050(p=0.465) | |  |
| 3. chronic emptiness | | 0.355(p<0.001) | | 0.373(p<0.001) | | | | 0.564(p<0.001) | | 0.341(p<0.001) | |  |
| 4. stress-related paranoia/ dissociation | | 0.551(p<0.001) | | 0.296(p<0.001) | | | | 0.635(p<0.001) | | 0.250(p=0.001) | |  |
| 5. serious identity disturbance | | 0.556(p<0.001) | | 0.286(p<0.001) | | | | 0.687(p<0.001) | | 0.360(p<0.001) | |  |
| 6. frantic efforts to avoid abandonment | | 0.566(p<0.001) | | -0.015(p=0.856) | | | | 0.510(p<0.001) | | -0.050(p=0.529) | |  |
| 7. self-destructive efforts | | 0.399(p<0.001) | | 0.128(p=0.077) | | | | 0.486(p<0.001) | | 0.116(p=0.184) | |  |
| 8. other impulsivity | | 0.593(p<0.001) | | -0.512(p<0.001) | | | | 0.620(p<0.001) | | -0.385(p<0.001) | |  |
| 9. stormy relationships | | 0.616(p<0.001) | | -0.112(p=0.126) | | | | 0.669(p<0.001) | | -0.252(p=0.001) | |  |
| Table A5-4: Item factor loading for ZAN-BPD (bi-factor 1 at 24 and 52 weeks and for overall data) | | | | | | | | | | | | |
| Item | 24 week | | | | | 52 week | | | Overall loading MI | | | |
|  | Factor G | | Factor 1 | | | Factor G | Factor 1 | | Factor G | | Factor 1 | |
| 1. chronic angry/frequent angry acts | 0.610(p<0.001) | | 0.354(p<0.001) | | | 0.717(p<0.001) | -0.290(p<0.001) | | 0.604(p<0.001) | | -0.301(p<0.001) | |
| 2. affective instability | 0.696(p<0.001) | | 0.015(p=0.859) | | | 0.725(p<0.001) | -0.104(p=0.125) | | 0.619(p<0.001) | | 0.007(p=0.927) | |
| 3. chronic emptiness | 0.587(p<0.001) | | -0.330(p<0.001) | | | 0.605(p<0.001) | 0.366(p<0.001) | | 0.427(p<0.001) | | 0.385(p<0.001) | |
| 4. stress-related paranoia/ dissociation | 0.690(p<0.001) | | -0.262(p=0.001) | | | 0.655(p<0.001) | 0.120(p=0.123) | | 0.533(p<0.001) | | 0.266(p<0.001) | |
| 5. serious identity disturbance | 0.745(p<0.001) | | -0.418(p<0.001) | | | 0.728(p<0.001) | 0.357(p<0.001) | | 0.562(p<0.001) | | 0.377(p<0.001) | |
| 6. frantic efforts to avoid abandonment | 0.556(p<0.001) | | 0.105(p=0.240) | | | 0.630(p<0.001) | -0.046(p=0.599) | | 0.510(p<0.001) | | -0.046(p=0.519) | |
| 7. self-destructive efforts | 0.421(p<0.001) | | -0.149(p=0.094) | | | 0.513(p<0.001) | 0.251(p=0.005) | | 0.388(p<0.001) | | 0.163(p=0.016) | |
| 8. other impulsivity | 0.630(p<0.001) | | 0.519(p<0.001) | | | 0.692(p<0.001) | -0.428(p<0.001) | | 0.637(p<0.001) | | -0.448(p<0.001) | |
| 9. stormy relationships | 0.632(p<0.001) | | 0.217(p=0.006) | | | 0.662(p<0.001) | -0.120(p=0.151) | | 0.603(p<0.001) | | -0.158(p=0.044) | |

| Table A6-1: Item factor loading for ZAN-BPD (three factors at baseline) | | | |
| --- | --- | --- | --- |
| Item | Baseline | | |
|  | Factor 1 | Factor 2 | Factor 3 |
| 1. chronic angry/frequent angry acts | 0.107(p=0.424) | 0.703(p<0.001) | -0.004(p=0.940) |
| 2. affective instability | 0.514(p<0.001) | 0.336(p=0.001) | -0.004(p=0.923) |
| 3. chronic emptiness | 0.472(p<0.001) | -0.118(p=0.256) | -0.001(p=0.993) |
| 4. stress-related paranoia/ dissociation | 0.529(p<0.001) | 0.010(p=0.408) | 0.313(p=0.008) |
| 5. serious identity disturbance | 0.601(p<0.001) | -0.055(p=0.551) | 0.227(p=0.061) |
| 6. frantic efforts to avoid abandonment | 0.236(p=0.031) | 0.165(p=0.174) | 0.367(p=0.002) |
| 7. self-destructive efforts | 0.475(p=0.001) | 0.026(p=0.647) | -0.225(p=0.108) |
| 8. other impulsivity | -0.006(p=0.599) | 0.605(p<0.001) | 0.120(p=0.305) |
| 9. stormy relationships | -0.001(p=0.754) | 0.492(p=0.001) | 0.611(p<0.001) |

| Table A6-2: Item factor loading for ZAN-BPD (three factors at 12 weeks) | | | |
| --- | --- | --- | --- |
| Item | 12 week | | |
|  | Factor 1 | Factor 2 | Factor 3 |
| 1. chronic angry/frequent angry acts | 0.054(p=0.714) | 0.698(p<0.001) | 0.004(p=0.951) |
| 2. affective instability | 0.396(p=0.004) | 0.413(p=0.001) | 0.002(p=0.959) |
| 3. chronic emptiness | 0.692(p<0.001) | -0.053(p=0.815) | -0.004(p=0.951) |
| 4. stress-related paranoia/ dissociation | 0.649(p<0.001) | -0.009(p=0.691) | 0.280(p=0.017) |
| 5. serious identity disturbance | 0.689(p<0.001) | 0.020(p=0.784) | 0.142(p=0.199) |
| 6. frantic efforts to avoid abandonment | 0.008(p=0.817) | 0.279(p=0.629) | 0.624(p<0.001) |
| 7. self-destructive efforts | 0.415(p=0.027) | 0.217(p=0.531) | -0.177(p=0.189) |
| 8. other impulsivity | 0.004(p=0.973) | 0.683(p<0.001) | -0.013(p=0.902) |
| 9. stormy relationships | -0.007(p=0.852) | 0.647(p=0.054) | 0.366(p=0.001) |

| Table A6-3: Item factor loading for ZAN-BPD (three factors at 24 weeks) | | | |
| --- | --- | --- | --- |
| Item | 24 week | | |
|  | Factor 1 | Factor 2 | Factor 3 |
| 1. chronic angry/frequent angry acts | 1.029(p<0.001) | 0.002(p=0.793) | -0.323(p=0.221) |
| 2. affective instability | 0.398(p<0.001) | 0.407(p<0.001) | 0.061(p=0.650) |
| 3. chronic emptiness | 0.063(p=0.575) | 0.614(p<0.001) | -0.005(p=0.937) |
| 4. stress-related paranoia/ dissociation | 0.007(p=0.500) | 0.677(p<0.001) | 0.129(p=0.356) |
| 5. serious identity disturbance | -0.163(p=0.244) | 0.955(p<0.001) | -0.016(p=0.588) |
| 6. frantic efforts to avoid abandonment | 0.006(p=0.439) | 0.003(p=0.788) | 1.023(p=0.020) |
| 7. self-destructive efforts | 0.144(p=0.208) | 0.272(p=0.028) | 0.044(p=0.712) |
| 8. other impulsivity | 0.730(p<0.001) | -0.090(p=0.430) | -0.022(p=0.754) |
| 9. stormy relationships | 0.415(p<0.001) | 0.162(p=0.256) | 0.178(p=0.338) |

| Table A6-4: Item factor loading for ZAN-BPD (three factors at 52 weeks) | | | |
| --- | --- | --- | --- |
| Item | 52 week | | |
|  | Factor 1 | Factor 2 | Factor 3 |
| 1. chronic angry/frequent angry acts | 0.946(p<0.001) | -0.141(p=0.441) | 0.002(p=0.960) |
| 2. affective instability | 0.682(p<0.001) | 0.184(p=0.154) | 0.007(p=0.903) |
| 3. chronic emptiness | 0.082(p=0.488) | 0.684(p<0.001) | 0.002(p=0.962) |
| 4. stress-related paranoia/ dissociation | 0.281(p=0.015) | 0.325(p=0.005) | 0.144(p=0.149) |
| 5. serious identity disturbance | -0.008(p=0.391) | 0.788(p<0.001) | 0.200(p=0.134) |
| 6. frantic efforts to avoid abandonment | 0.176(p=0.214) | 0.184(p=0.101) | 0.393(p=0.029) |
| 7. self-destructive efforts | 0.383(p=0.007) | 0.403(p=0.003) | -0.162(p=0.190) |
| 8. other impulsivity | 0.560(p<0.001) | 0.003(p=0.787) | 0.224(p=0.041) |
| 9. stormy relationships | 0.005(p=0.589) | -0.004(p=0.670) | 1.026(p<0.001) |
| Table A6-5: Item factor loading for ZAN-BPD (three factors at baseline) | | | |
| Item | baseline | | |
|  | Factor 1 | Factor 2 | Factor 3 |
| 1. chronic angry/frequent angry acts | 0.185(p=0.029) | 0.512(p<0.001) | -0.021(p=0.673) |
| 2. affective instability | 0.537(p<0.001) | 0.167(p=0.149) | 0.125(p=0.129) |
| 3. chronic emptiness | 0.312(p=0.003) | -0.001(p=0.940) | 0.431(p<0.001) |
| 4. stress-related paranoia/ dissociation | 0.780(p<0.001) | -0.243(p=0.016) | -0.016(p=0.372) |
| 5. serious identity disturbance | 0.714(p<0.001) | -0.165(p=0.152) | 0.064(p=0.522) |
| 6. frantic efforts to avoid abandonment | 0.629(p<0.001) | -0.005(p=0.937) | -0.196(p=0.037) |
| 7. self-destructive efforts | 0.000(p=0.946) | 0.394(p=0.001) | 0.527(p<0.001) |
| 8. other impulsivity | 0.010(p=0.596) | 0.731(p<0.001) | -0.004(p=0.930) |
| 9. stormy relationships | 0.685(p<0.001) | 0.016(p=0.791) | -0.348(p<0.001) |

| Table A6-6: Item factor loading for ZAN-BPD (three factors at 12 weeks) | | | |
| --- | --- | --- | --- |
| Item | 12 week | | |
|  | Factor 1 | Factor 2 | Factor 3 |
| 1. chronic angry/frequent angry acts | 0.009(p=0.905) | 0.657(p<0.001) | -0.194(p=0.061) |
| 2. affective instability | 0.319(p<0.001) | 0.436(p<0.001) | -0.004(p=0.820) |
| 3. chronic emptiness | 0.758(p<0.001) | -0.119(p=0.246) | -0.007(p=0.562) |
| 4. stress-related paranoia/ dissociation | 0.533(p<0.001) | 0.124(p=0.306) | 0.240(p<0.001) |
| 5. serious identity disturbance | 0.737(p<0.001) | -0.007(p=0.527) | 0.193(p=0.029) |
| 6. frantic efforts to avoid abandonment | 0.023(p=0.519) | 0.454(p<0.001) | 0.342(p<0.001) |
| 7. self-destructive efforts | 0.624(p<0.001) | 0.005(p=0.241) | -0.410(p<0.001) |
| 8. other impulsivity | 0.004(p=0.928) | 0.732(p<0.001) | -0.243(p=0.077) |
| 9. stormy relationships | -0.038(p=0.644) | 0.743(p<0.001) | 0.147(p=0.229) |
| Table A6-7: Item factor loading for ZAN-BPD (three factors at 24 weeks) | | | |
| Item | 24 week | | |
|  | Factor 1 | Factor 2 | Factor 3 |
| 1. chronic angry/frequent angry acts | 0.923(p<0.001) | -0.359(p=0.020) | -0.011(p=0.810) |
| 2. affective instability | 0.561(p<0.001) | 0.174(p=0.102) | 0.017(p=0.759) |
| 3. chronic emptiness | 0.001(p=0.957) | 0.510(p<0.001) | 0.330(p=0.002) |
| 4. stress-related paranoia/ dissociation | 0.183(p=0.286) | 0.630(p<0.001) | 0.004(p=0.868) |
| 5. serious identity disturbance | 0.007(p=0.448) | 0.777(p<0.001) | 0.209(p=0.048) |
| 6. frantic efforts to avoid abandonment | 0.577(p<0.001) | 0.093(p=0.448) | -0.161(p=0.057) |
| 7. self-destructive efforts | 0.134(p=0.651) | 0.020(p=0.623) | 0.485(p=0.001) |
| 8. other impulsivity | 1.142(p<0.001) | -0.617(p=0.002) | 0.008(p=0.715) |
| 9. stormy relationships | 0.810(p<0.001) | 0.004(p=0.716) | -0.298(p=0.006) |
| Table A6-8: Item factor loading for ZAN-BPD (three factors at 52 weeks) | | | |
| Item | 52 week | | |
|  | Factor 1 | Factor 2 | Factor 3 |
| 1. chronic angry/frequent angry acts | 0.711(p<0.001) | 0.153(p=0.115) | -0.021(p=0.525) |
| 2. affective instability | 0.500(p<0.001) | 0.328(p<0.001) | 0.030(p=0.620) |
| 3. chronic emptiness | -0.081(p=0.398) | 0.784(p<0.001) | -0.009(p=0.541) |
| 4. stress-related paranoia/ dissociation | 0.215(p=0.021) | 0.380(p<0.001) | 0.244(p=0.001) |
| 5. serious identity disturbance | -0.004(p=0.513) | 0.673(p<0.001) | 0.249(p=0.003) |
| 6. frantic efforts to avoid abandonment | 0.369(p=0.002) | 0.044(p=0.717) | 0.470(p<0.001) |
| 7. self-destructive efforts | 0.006(p=0.200) | 0.835(p<0.001) | -0.368(p=0.002) |
| 8. other impulsivity | 0.851(p<0.001) | -0.009(p=0.672) | -0.019(p=0.760) |
| 9. stormy relationships | 0.484(p<0.001) | -0.017(p=0.070) | 0.455(p<0.001) |

| Table A6-9: Item factor loading for ZAN-BPD (three factors for overall data) | | | | | | | |  |
| --- | --- | --- | --- | --- | --- | --- | --- | --- |
| Item | | Overall loading MI | | | | | |  |
|  |  | Factor 1 | | Factor 2 | | Factor 3 | |  |
| 1. chronic angry/frequent angry acts | | 0.619(p<0.001) | | 0.069(p=0.127) | | -0.009(p=0.536) | |  |
| 2. affective instability | | 0.354(p<0.001) | | 0.285(p<0.001) | | 0.147(p=0.002) | |  |
| 3. chronic emptiness | | -0.147(p=0.016) | | 0.617(p<0.001) | | -0.005(p=0.298) | |  |
| 4. stress-related paranoia/ dissociation | | 0.082(p=0.231) | | 0.383(p<0.001) | | 0.336(p<0.001) | |  |
| 5. serious identity disturbance | | -0.019(p=0.141) | | 0.542(p<0.001) | | 0.265(p<0.001) | |  |
| 6. frantic efforts to avoid abandonment | | 0.387(p<0.001) | | 0.048(p=0.389) | | 0.371(p<0.001) | |  |
| 7. self-destructive efforts | | 0.011(p=0.051) | | 0.521(p<0.001) | | -0.295(p<0.001) | |  |
| 8. other impulsivity | | 0.760(p<0.001) | | -0.003(p=0.652) | | -0.090(p=0.132) | |  |
| 9. stormy relationships | | 0.564(p<0.001) | | -0.015(p=0.034) | | 0.374(p<0.001) | |  |
| Table A7-1: Item factor loading for ZAN-BPD (four factors at baseline) | | | | | | | | |
| Item | baseline | | | | | | | |
|  | Factor 1 | | Factor 2 | | Factor 3 | | Factor 4 | |
| 1. chronic angry/frequent angry acts | 0.088(p=0.203) | | 0.660(p<0.001) | | 0.042(p=0.428) | | -0.051(p=0.192) | |
| 2. affective instability | 0.316(p=0.003) | | 0.238(p=0.005) | | 0.306(p=0.001) | | 0.101(p=0.194) | |
| 3. chronic emptiness | 0.648(p<0.001) | | 0.038(p=0.362) | | -0.058(p=0.149) | | -0.021(p=0.739) | |
| 4. stress-related paranoia/ dissociation | 0.372(p<0.001) | | -0.021(p=0.603) | | 0.486(p<0.001) | | -0.054(p=0.307) | |
| 5. serious identity disturbance | 0.362(p<0.001) | | -0.035(p=0.516) | | 0.450(p<0.001) | | 0.050(p=0.324) | |
| 6. frantic efforts to avoid abandonment | -0.044(p=0.493) | | 0.054(p=0.396) | | 0.602(p<0.001) | | 0.175(p=0.134) | |
| 7. self-destructive efforts | 0.251(p=0.203) | | -0.008(p=0.751) | | 0.006(p=0.816) | | 0.462(p=0.001) | |
| 8. other impulsivity | -0.050(p=0.222) | | 0.749(p<0.001) | | -0.020(p=0.501) | | 0.080(p=0.245) | |
| 9. stormy relationships | -0.006(p=0.796) | | 0.341(p<0.001) | | 0.544(p<0.001) | | -0.117(p=0.249) | |

| Table A7-2: Item factor loading for ZAN-BPD (four factors at 12 weeks) | | | | |
| --- | --- | --- | --- | --- |
| Item | 12 week | | | |
|  | Factor 1 | Factor 2 | Factor 3 | Factor 4 |
| 1. chronic angry/frequent angry acts | 0.093(p=0.318) | 0.706(p<0.001) | -0.077(p=0.339) | -0.008(p=0.771) |
| 2. affective instability | 0.178(p=0.068) | 0.292(p=0.001) | 0.222(p=0.025) | 0.195(p=0.028) |
| 3. chronic emptiness | 0.686(p<0.001) | 0.070(p=0.321) | -0.038(p=0.521) | 0.029(p=0.610) |
| 4. stress-related paranoia/ dissociation | 0.488(p<0.001) | 0.011(p=0.808) | 0.338(p=0.002) | -0.004(p=0.919) |
| 5. serious identity disturbance | 0.647(p<0.001) | -0.029(p=0.507) | 0.266(p=0.037) | 0.024(p=0.590) |
| 6. frantic efforts to avoid abandonment | -0.002(p=0.963) | 0.048(p=0.430) | 0.590(p<0.001) | 0.010(p=0.818) |
| 7. self-destructive efforts | 0.062(p=0.342) | 0.014(p=0.574) | -0.036(p=0.190) | 0.612(p<0.001) |
| 8. other impulsivity | -0.097(p=0.219) | 0.652(p<0.001) | 0.046(p=0.144) | 0.194(p=0.046) |
| 9. stormy relationships | 0.013(p=0.678) | 0.466(p<0.001) | 0.407(p<0.001) | -0.034(p=0.368) |
| Table A7-3: Item factor loading for ZAN-BPD (four factors at 24 weeks) | | | | |
| Item | 24 week | | | |
|  | Factor 1 | Factor 2 | Factor 3 | Factor 4 |
| 1. chronic angry/frequent angry acts | 0.042(p=0.411) | 0.673(p<0.001) | 0.063(p=0.412) | 0.000(p=0.999) |
| 2. affective instability | 0.267(p=0.010) | 0.267(p<0.001) | 0.317(p=0.006) | 0.112(p=0.149) |
| 3. chronic emptiness | 0.652(p<0.001) | 0.155(p=0.146) | -0.069(p=0.483) | 0.007(p=0.866) |
| 4. stress-related paranoia/ dissociation | 0.499(p<0.001) | -0.031(p=0.563) | 0.424(p=0.007) | -0.01(p=0.792) |
| 5. serious identity disturbance | 0.723(p<0.001) | -0.014(p=0.728) | 0.241(p=0.206) | 0.001(p=0.990) |
| 6. frantic efforts to avoid abandonment | -0.041(p=0.467) | 0.024(p=0.434) | 0.641(p<0.001) | 0.22(p=0.126) |
| 7. self-destructive efforts | 0.214(p=0.292) | 0.015(p=0.474) | 0.004(p=0.868) | 0.608(p<0.001) |
| 8. other impulsivity | -0.065(p=0.187) | 0.869(p<0.001) | -0.018(p=0.421) | 0.034(p=0.398) |
| 9. stormy relationships | 0.031(p=0.291) | 0.373(p<0.001) | 0.507(p<0.001) | -0.159(p=0.096) |
| Table A7-4: Item factor loading for ZAN-BPD (four factors at 52 weeks) | | | | |
| Item | 52 week | | | |
|  | Factor 1 | Factor 2 | Factor 3 | Factor 4 |
| 1. chronic angry/frequent angry acts | 0.705(p<0.001) | 0.001(p=0.991) | 0.049(p=0.514) | 0.113(p=0.202) |
| 2. affective instability | 0.426(p<0.001) | 0.022(p=0.659) | 0.241(p=0.018) | 0.220(p=0.017) |
| 3. chronic emptiness | 0.100(p=0.355) | 0.729(p<0.001) | 0.018(p=0.411) | 0.029(p=0.620) |
| 4. stress-related paranoia/ dissociation | 0.080(p=0.369) | 0.142(p=0.208) | 0.484(p<0.001) | 0.062(p=0.467) |
| 5. serious identity disturbance | -0.053(p=0.059) | 0.474(p<0.001) | 0.477(p<0.001) | 0.008(p=0.839) |
| 6. frantic efforts to avoid abandonment | 0.011(p=0.503) | -0.240(p=0.104) | 0.813(p<0.001) | 0.019(p=0.523) |
| 7. self-destructive efforts | 0.047(p=0.237) | 0.157(p=0.513) | 0.014(p=0.463) | 0.677(p<0.001) |
| 8. other impulsivity | 0.856(p<0.001) | -0.021(p=0.688) | -0.024(p=0.515) | -0.022(p=0.416) |
| 9. stormy relationships | 0.297(p=0.011) | 0.032(p=0.037) | 0.562(p<0.001) | -0.274(p<0.001) |

| Table A7-5: Item factor loading for ZAN-BPD (four factors for overall data) | | | | | | | | |
| --- | --- | --- | --- | --- | --- | --- | --- | --- |
| Item | Overall loading MI | | | | | | | |
|  | Factor 1 | | Factor 2 | | Factor 3 | | Factor 4 | |
| 1. chronic angry/frequent angry acts | 0.653(p<0.001) | | 0.025(p=0.477) | | 0.051(p=0.058) | | -0.043(p=0.160) | |
| 2. affective instability | 0.283(p<0.001) | | 0.321(p<0.001) | | 0.140(p=0.005) | | 0.098(p=0.015) | |
| 3. chronic emptiness | 0.100(p=0.072) | | -0.018(p=0.461) | | 0.562(p<0.001) | | 0.043(p=0.222) | |
| 4. stress-related paranoia/ dissociation | 0.002(p=0.944) | | 0.494(p<0.001) | | 0.317(p<0.001) | | -0.056(p=0.117) | |
| 5. serious identity disturbance | -0.044(p=0.175) | | 0.449(p<0.001) | | 0.413(p<0.001) | | 0.045(p=0.096) | |
| 6. frantic efforts to avoid abandonment | 0.025(p=0.132) | | 0.699(p<0.001) | | -0.127(p=0.087) | | 0.077(p=0.169) | |
| 7. self-destructive efforts | 0.021(p=0.230) | | 0.030(p=0.070) | | 0.047(p=0.212) | | 0.637(p<0.001) | |
| 8. other impulsivity | 0.755(p<0.001) | | -0.005(p=0.801) | | -0.085(p=0.022) | | 0.053(p=0.083) | |
| 9. stormy relationships | 0.352(p<0.001) | | 0.535(p<0.001) | | 0.004(p=0.795) | | -0.210(p=0.002) | |
| Table A8-1: Item factor loading for ZAN-BPD (bi(2)-factor at baseline) | | | | | | | |  |
| Item | | Baseline | | | | | |  |
|  |  | Factor G | | Factor 1 | | Factor 2 | |  |
| 1. chronic angry/frequent angry acts | | 0.606(p<0.001) | | -0.437(p=0.003) | | -0.019(p=0.589) | |  |
| 2. affective instability | | 0.674(p<0.001) | | -0.069(p=0.624) | | -0.191(p=0.587) | |  |
| 3. chronic emptiness | | 0.306(p=0.048) | | 0.222(p=0.160) | | -0.188(p=0.622) | |  |
| 4. stress-related paranoia/ dissociation | | 0.619(p<0.001) | | 0.320(p=0.012) | | 0.046(p=0.933) | |  |
| 5. serious identity disturbance | | 0.584(p<0.001) | | 0.339(p=0.057) | | -0.053(p=0.928) | |  |
| 6. frantic efforts to avoid abandonment | | 0.518(p<0.001) | | 0.156(p=0.146) | | 0.209(p=0.600) | |  |
| 7. self-destructive efforts | | 0.293(p=0.188) | | 0.008(p=0.840) | | -0.364(p=0.098) | |  |
| 8. other impulsivity | | 0.507(p<0.001) | | -0.341(p=0.006) | | 0.120(p=0.355) | |  |
| 9. stormy relationships | | 0.696(p=0.015) | | -0.005(p=0.842) | | 0.508(p=0.243) | |  |
| A8-2: Item factor loading for ZAN-BPD (bi(2)-factor at 12 weeks) | | | | | | | |  |
| Item | | 12 week | | | | | |  |
|  |  | Factor G | | Factor 1 | | Factor 2 | |  |
| 1. chronic angry/frequent angry acts | | 0.632(p<0.001) | | 0.362(p=0.011) | | -0.007(p=0.908) | |  |
| 2. affective instability | | 0.699(p<0.001) | | 0.064(p=0.647) | | -0.074(p=0.458) | |  |
| 3. chronic emptiness | | 0.574(p<0.001) | | -0.314(p=0.041) | | -0.138(p=0.322) | |  |
| 4. stress-related paranoia/ dissociation | | 0.657(p<0.001) | | -0.347(p=0.023) | | 0.126(p=0.326) | |  |
| 5. serious identity disturbance | | 0.677(p<0.001) | | -0.311(p=0.041) | | -0.007(p=0.811) | |  |
| 6. frantic efforts to avoid abandonment | | 0.426(p<0.001) | | -0.014(p=0.405) | | 0.560(p<0.001) | |  |
| 7. self-destructive efforts | | 0.500(p<0.001) | | -0.004(p=0.866) | | -0.240(p=0.073) | |  |
| 8. other impulsivity | | 0.570(p<0.001) | | 0.379(p=0.001) | | -0.012(p=0.855) | |  |
| 9. stormy relationships | | 0.642(p<0.001) | | 0.264(p=0.027) | | 0.331(p=0.002) | |  |

| A8-3: Item factor loading for ZAN-BPD (bi(2)-factor at 24 weeks) | | | |
| --- | --- | --- | --- |
| Item | 24 week | | |
|  | Factor G | Factor 1 | Factor 2 |
| 1. chronic angry/frequent angry acts | 0.813(p<0.001) | -0.005(p=0.607) | -0.401(p=0.202) |
| 2. affective instability | 0.662(p<0.001) | 0.334(p=0.001) | -0.019(p=0.682) |
| 3. chronic emptiness | 0.405(p<0.001) | 0.508(p<0.001) | -0.024(p=0.681) |
| 4. stress-related paranoia/ dissociation | 0.471(p<0.001) | 0.560(p<0.001) | 0.087(p=0.267) |
| 5. serious identity disturbance | 0.368(p<0.001) | 0.792(p<0.001) | -0.005(p=0.667) |
| 6. frantic efforts to avoid abandonment | 0.659(p<0.001) | -0.002(p=0.657) | 0.788(p=0.175) |
| 7. self-destructive efforts | 0.324(p<0.001) | 0.224(p=0.032) | 0.008(p=0.910) |
| 8. other impulsivity | 0.657(p<0.001) | -0.079(p=0.399) | -0.123(p=0.382) |
| 9. stormy relationships | 0.615(p<0.001) | 0.130(p=0.267) | 0.073(p=0.500) |
| A8-4: Item factor loading for ZAN-BPD (bi(2)-factor at 52 weeks) | | | |
| Item | 52 week | | |
|  | Factor G | Factor 1 | Factor 2 |
| 1. chronic angry/frequent angry acts | 0.703(p<0.001) | 0.537(p<0.001) | -0.002(p=0.753) |
| 2. affective instability | 0.753(p<0.001) | 0.251(p=0.021) | -0.048(p=0.500) |
| 3. chronic emptiness | 0.655(p<0.001) | -0.286(p=0.011) | -0.124(p=0.192) |
| 4. stress-related paranoia/ dissociation | 0.607(p<0.001) | -0.017(p=0.872) | 0.049(p=0.470) |
| 5. serious identity disturbance | 0.784(p<0.001) | -0.382(p=0.005) | 0.019(p=0.201) |
| 6. frantic efforts to avoid abandonment | 0.543(p<0.001) | -0.003(p=0.968) | 0.278(p=0.014) |
| 7. self-destructive efforts | 0.580(p<0.001) | -0.002(p=0.963) | -0.214(p=0.075) |
| 8. other impulsivity | 0.622(p<0.001) | 0.275(p=0.003) | 0.163(p=0.046) |
| 9. stormy relationships | 0.611(p<0.001) | -0.001(p=0.915) | 0.826(p=0.002) |

| A8-5: Item factor loading for ZAN-BPD (bi(2)-factor overall data) | | | | | | | |  |
| --- | --- | --- | --- | --- | --- | --- | --- | --- |
| Item | | Overall loading MI | | | | | |  |
|  |  | Factor G | | Factor 1 | | Factor 2 | |  |
| 1. chronic angry/frequent angry acts | | 0.556(p<0.001) | | -0.329(p<0.001) | | 0.009(p=0.742) | |  |
| 2. affective instability | | 0.614(p<0.001) | | -0.012(p=0.828) | | 0.019(p=0.584) | |  |
| 3. chronic emptiness | | 0.393(p<0.001) | | 0.286(p<0.001) | | -0.238(p<0.001) | |  |
| 4. stress-related paranoia/ dissociation | | 0.588(p<0.001) | | 0.289(p<0.001) | | 0.093(p=0.039) | |  |
| 5. serious identity disturbance | | 0.596(p<0.001) | | 0.355(p<0.001) | | -0.019(p=0.219) | |  |
| 6. frantic efforts to avoid abandonment | | 0.576(p<0.001) | | 0.027(p=0.378) | | 0.259(p<0.001) | |  |
| 7. self-destructive efforts | | 0.269(p<0.001) | | -0.012(p=0.089) | | -0.388(p<0.001) | |  |
| 8. other impulsivity | | 0.563(p<0.001) | | -0.482(p<0.001) | | -0.010(p=0.549) | |  |
| 9. stormy relationships | | 0.670(p<0.001) | | -0.092(p=0.250) | | 0.296(p<0.001) | |  |
| A9-1: Item factor loading for ZAN-BPD (bi(3)-factor at 12 weeks, data at baseline not convergent.) | | | | | | | | |
| Item | 12 week | | | | | | | |
|  | Factor G | | Factor 1 | | Factor 2 | | Factor 3 | |
| 1. chronic angry/frequent angry acts | 0.013(p=0.953) | | 0.673(p=0.064) | | -0.372(p=0.129) | | -0.007(p=0.913) | |
| 2. affective instability | -0.020(p=0.956) | | 0.702(p<0.001) | | 0.022(p=0.905) | | -0.007(p=0.936) | |
| 3. chronic emptiness | 0.067(p=0.939) | | 0.512(p=0.016) | | 0.183(p=0.422) | | -0.125(p=0.369) | |
| 4. stress-related paranoia/ dissociation | 2.088(p=0.857) | | 0.617(p=0.657) | | 0.000(p=0.997) | | 0.001(p=0.978) | |
| 5. serious identity disturbance | -0.018(p=0.982) | | 0.682(p=0.203) | | 0.556(p=0.070) | | 0.001(p=0.976) | |
| 6. frantic efforts to avoid abandonment | 0.043(p=0.947) | | 0.380(p=0.293) | | 0.131(p=0.364) | | 0.387(p=0.013) | |
| 7. self-destructive efforts | -0.012(p=0.959) | | 0.514(p=0.007) | | -0.002(p=0.988) | | -0.245(p=0.105) | |
| 8. other impulsivity | -0.037(p=0.394) | | 0.602(p=0.022) | | -0.258(p=0.212) | | 0.029(p=0.763) | |
| 9. stormy relationships | -0.022(p=0.944) | | 0.659(p=0.104) | | -0.049(p=0.566) | | 0.540(p=0.045) | |

| A9-2: Item factor loading for ZAN-BPD (bi(3)-factor at 24 weeks) | | | | |
| --- | --- | --- | --- | --- |
| Item | 24 week | | | |
|  | Factor G | Factor 1 | Factor 2 | Factor 3 |
| 1. chronic angry/frequent angry acts | 0.686(p<0.001) | 0.708(p=0.046) | -0.024(p=0.609) | -0.024(p=0.521) |
| 2. affective instability | 0.730(p<0.001) | 0.062(p=0.389) | 0.124(p=0.507) | 0.090(p=0.425) |
| 3. chronic emptiness | 0.528(p<0.001) | 0.012(p=0.824) | 0.330(p=0.097) | 0.195(p=0.169) |
| 4. stress-related paranoia/ dissociation | 0.625(p<0.001) | -0.099(p=0.141) | 0.363(p=0.057) | -0.006(p=0.938) |
| 5. serious identity disturbance | 0.558(p=0.001) | 0.002(p=0.942) | 0.737(p<0.001) | -0.015(p=0.677) |
| 6. frantic efforts to avoid abandonment | 0.677(p<0.001) | -0.380(p=0.157) | -0.142(p=0.432) | -0.031(p=0.567) |
| 7. self-destructive efforts | 0.407(p<0.001) | -0.029(p=0.696) | 0.009(p=0.918) | 0.363(p=0.130) |
| 8. other impulsivity | 0.571(p<0.001) | 0.228(p=0.053) | -0.184(p=0.111) | 0.021(p=0.788) |
| 9. stormy relationships | 0.681(p<0.001) | -0.002(p=0.938) | 0.022(p=0.733) | -0.462(p=0.137) |
| A9-3: Item factor loading for ZAN-BPD (bi(3)-factor at 52 weeks | | | | |
| Item | 52 week | | | |
|  | Factor G | Factor 1 | Factor 2 | Factor 3 |
| 1. chronic angry/frequent angry acts | 0.601(p<0.001) | 0.259(p=0.110) | -0.011(p=0.824) | 0.411(p=0.123) |
| 2. affective instability | 0.774(p<0.001) | 0.584(p=0.171) | -0.008(p=0.827) | -0.020(p=0.754) |
| 3. chronic emptiness | 0.685(p<0.001) | -0.190(p=0.532) | -0.144(p=0.229) | -0.040(p=0.699) |
| 4. stress-related paranoia/ dissociation | 0.601(p<0.001) | 0.090(p=0.573) | 0.098(p=0.284) | -0.041(p=0.733) |
| 5. serious identity disturbance | 0.828(p<0.001) | -0.274(p=0.453) | 0.001(p=0.990) | -0.072(p=0.637) |
| 6. frantic efforts to avoid abandonment | 0.532(p<0.001) | 0.043(p=0.671) | 0.328(p=0.027) | -0.007(p=0.951) |
| 7. self-destructive efforts | 0.569(p<0.001) | -0.015(p=0.867) | -0.268(p=0.058) | 0.168(p=0.230) |
| 8. other impulsivity | 0.569(p<0.001) | -0.019(p=0.798) | 0.048(p=0.678) | 0.586(p=0.008) |
| 9. stormy relationships | 0.586(p<0.001) | -0.020(p=0.517) | 0.752(p=0.003) | 0.027(p=0.662) |

| A10-1: Item factor loading for ZAN-BPD (bi(4)-factor at 24 weeks, data at baseline and 12 weeks not convergent) | | | | | |
| --- | --- | --- | --- | --- | --- |
| Item | 24 week | | | | |
|  | Factor G | Factor 1 | Factor 2 | Factor 3 | Factor 4 |
| 1. chronic angry/frequent angry acts | 0.704(p=0.166) | 0.619(p=0.667) | -0.065(p=0.931) | -0.043(p=0.691) | -0.004(p=0.959) |
| 2. affective instability | 0.724(p<0.001) | 0.050(p=0.823) | 0.040(p=0.945) | 0.108(p=0.750) | 0.062(p=0.664) |
| 3. chronic emptiness | 0.525(p=0.067) | -0.027(p=0.835) | 0.026(p=0.880) | 0.476(p=0.582) | 0.039(p=0.951) |
| 4. stress-related paranoia/ dissociation | 0.641(p=0.127) | -0.054(p=0.765) | 0.402(p=0.577) | 0.002(p=0.996) | 0.066(p=0.682) |
| 5. serious identity disturbance | 0.581(p=0.302) | 0.011(p=0.934) | 0.497(p=0.489) | 0.263(p=0.829) | -0.034(p=0.848) |
| 6. frantic efforts to avoid abandonment | 0.682(p<0.001) | -0.506(p=0.680) | -0.208(p=0.859) | -0.024(p=0.879) | -0.009(p=0.938) |
| 7. self-destructive efforts | 0.391(p=0.007) | -0.001(p=0.993) | 0.013(p=0.912) | -0.008(p=0.962) | 0.567(p=0.735) |
| 8. other impulsivity | 0.573(p=0.148) | 0.172(p=0.720) | -0.279(p=0.294) | 0.044(p=0.859) | -0.015(p=0.931) |
| 9. stormy relationships | 0.680(p<0.001) | -0.013(p=0.952) | 0.067(p=0.933) | -0.147(p=0.846) | -0.240(p=0.839) |
| A10-2: Item factor loading for ZAN-BPD (bi(4)-factor at 52 weeks) | | | | | |
| Item | 52 week | | | | |
|  | Factor G | Factor 1 | Factor 2 | Factor 3 | Factor 4 |
| 1. chronic angry/frequent angry acts | 0.698(p=0.003) | 0.209(p=0.737) | -0.306(p=0.091) | -0.030(p=0.824) | 0.015(p=0.884) |
| 2. affective instability | 0.717(p=0.025) | 0.596(p=0.230) | -0.019(p=0.888) | 0.001(p=0.984) | 0.021(p=0.730) |
| 3. chronic emptiness | 0.586(p=0.002) | -0.001(p=0.988) | 0.307(p=0.352) | 0.023(p=0.839) | 0.226(p=0.456) |
| 4. stress-related paranoia/ dissociation | 0.568(p<0.001) | 0.222(p=0.123) | 0.170(p=0.195) | 0.020(p=0.945) | -0.088(p=0.791) |
| 5. serious identity disturbance | 0.775(p=0.002) | -0.042(p=0.813) | 0.502(p=0.341) | -0.071(p=0.787) | 0.003(p=0.907) |
| 6. frantic efforts to avoid abandonment | 0.556(p<0.001) | 0.002(p=0.987) | 0.003(p=0.965) | 0.587(p=0.745) | 0.003(p=0.983) |
| 7. self-destructive efforts | 0.534(p<0.001) | 0.017(p=0.930) | -0.002(p=0.991) | 0.017(p=0.938) | 0.424(p=0.068) |
| 8. other impulsivity | 0.747(p<0.001) | -0.131(p=0.851) | -0.379(p=0.412) | -0.019(p=0.814) | -0.020(p=0.708) |
| 9. stormy relationships | 0.673(p<0.001) | -0.006(p=0.958) | -0.007(p=0.950) | 0.238(p=0.845) | -0.384(p=0.413) |

| A10-3: Item factor loading for ZAN-BPD (bi(4)-factor for overall data) | | | | | |
| --- | --- | --- | --- | --- | --- |
| Item | Overall loading MI | | | | |
|  | Factor G | Factor 1 | Factor 2 | Factor 3 | Factor 4 |
| 1. chronic angry/frequent angry acts | 0.500(p<0.001) | 0.057(p=0.085) | -0.020(p=0.268) | 0.344(p<0.001) | 0.223(p=0.011) |
| 2. affective instability | 0.637(p<0.001) | -0.028(p=0.266) | 0.133(p=0.005) | 0.005(p=0.891) | 0.157(p=0.012) |
| 3. chronic emptiness | 0.405(p<0.001) | 0.418(p<0.001) | 0.039(p=0.196) | -0.038(p=0.153) | -0.029(p=0.434) |
| 4. stress-related paranoia/ dissociation | 0.652(p<0.001) | 0.044(p=0.346) | -0.058(p=0.158) | -0.192(p=0.001) | 0.028(p=0.412) |
| 5. serious identity disturbance | 0.609(p<0.001) | 0.120(p=0.329) | 0.003(p=0.873) | -0.032(p=0.390) | -0.243(p=0.005) |
| 6. frantic efforts to avoid abandonment | 0.611(p<0.001) | -0.360(p<0.001) | 0.034(p=0.157) | -0.054(p=0.130) | -0.029(p=0.292) |
| 7. self-destructive efforts | 0.278(p<0.001) | -0.004(p=0.684) | 0.622(p<0.001) | 0.015(p=0.160) | -0.017(p=0.176) |
| 8. other impulsivity | 0.448(p<0.001) | -0.029(p=0.255) | 0.013(p=0.517) | 0.571(p<0.001) | 0.022(p=0.269) |
| 9. stormy relationships | 0.652(p<0.001) | -0.171(p=0.054) | -0.241(p<0.001) | 0.191(p=0.006) | -0.023(p=0.201) |

**Appendix C**

Table A11-1 Summary of average model fittings(standard deviation, sd) and performance for 2-factor model and bi-1,2,3 factor models.

| model | χ^2^(sd),df | CFI(sd) | NNFI(sd) | RMSEA(sd) | Number of successful computations |
| --- | --- | --- | --- | --- | --- |
| 2-factor | 18.886(5.77),19 | .993(.012) | .987(.022) | .013(.016) | 9875 |
| Bi 1-factor | 18.889(5.78),19 | .993(.012) | .987(.022) | .013(.016) | 9883 |
| Bi 2-factor | 9.620(3.741),12 | .998(.006) | .993(.017) | .006(.013) | 7303 |
| Bi 3-factor | 3.048(1.74),6 | 1.000(.002) | .998(.010) | .002(.007) | 4234 |

MODEL RESULTS (2-factor model)

ESTIMATES S. E. M. S. E. 95% % Sig

Population Average Std. Dev. Average Cover Coeff

F1 BY

**ZBPD1** 0.668 0.5444 0.1168 0.1037 0.0289 0.740 **0.970**

**ZBPD2** 0.386 0.3366 0.1341 0.1195 0.0204 0.878 **0.765**

ZBPD3 -0.092 -0.0412 0.1079 0.1157 0.0142 0.777 0.047

ZBPD4 0.088 0.1002 0.1218 0.1110 0.0150 0.751 0.182

ZBPD5 0.001 0.0368 0.1064 0.0967 0.0126 0.922 0.078

**ZBPD6** 0.368 0.3316 0.1291 0.1147 0.0180 0.886 **0.772**

ZBPD7 0.092 0.1024 0.1265 0.1165 0.0161 0.856 0.198

**ZBPD8** 0.828 0.6322 0.1214 0.1140 0.0531 0.506 **0.985**

**ZBPD9** 0.532 0.4485 0.1234 0.1097 0.0222 0.842 **0.926**

F2 BY

ZBPD1 0.014 0.0381 0.1122 0.1030 0.0132 0.869 0.090

ZBPD2 0.333 0.2876 0.1378 0.1223 0.0211 0.873 0.647

**ZBPD3** 0.614 0.5169 0.1279 0.1160 0.0258 0.811 **0.975**

ZBPD4 0.548 0.4626 0.1253 0.1091 0.0230 0.840 **0.938**

**ZBPD5** 0.676 0.5461 0.1250 0.1079 0.0325 0.739 0.969

ZBPD6 0.222 0.2055 0.1347 0.1210 0.0184 0.853 0.443

**ZBPD7** 0.369 0.3350 0.1271 0.1134 0.0173 0.897 **0.795**

ZBPD8 -0.117 -0.0465 0.0987 0.1087 0.0147 0.627 0.032

ZBPD9 0.158 0.1491 0.1307 0.1188 0.0172 0.776 0.287

MODEL RESULTS (bi-1 factor model)

ESTIMATES S. E. M. S. E. 95% % Sig

Population Average Std. Dev. Average Cover Coeff

F1 BY

ZBPD1 0.604 0.4948 0.0716 0.0804 0.0170 0.565 **0.994**

ZBPD2 0.619 0.5280 0.0661 0.0630 0.0127 0.701 **0.998**

ZBPD3 0.427 0.3818 0.0821 0.0868 0.0088 0.818 **0.988**

ZBPD4 0.533 0.4663 0.0725 0.0775 0.0097 0.793 **0.995**

ZBPD5 0.562 0.4764 0.0785 0.0835 0.0135 0.688 **0.992**

ZBPD6 0.510 0.4550 0.0711 0.0741 0.0081 0.873 **0.996**

ZBPD7 0.388 0.3646 0.0782 0.0789 0.0067 0.914 **0.991**

ZBPD8 0.637 0.4945 0.0885 0.0910 0.0281 0.372 **0.991**

ZBPD9 0.603 0.5081 0.0678 0.0709 0.0136 0.658 **0.997**

F2 BY

**ZBPD1** -0.301 -0.2633 0.1095 0.1475 0.0134 0.928 **0.714**

ZBPD2 0.007 -0.0105 0.1016 0.1574 0.0106 0.940 0.057

**ZBPD3** 0.385 0.3236 0.1312 0.1528 0.0210 0.877 **0.825**

ZBPD4 0.266 0.2140 0.1068 0.1450 0.0141 0.919 0.560

**ZBPD5** 0.377 0.2969 0.1188 0.1573 0.0205 0.869 **0.775**

ZBPD6 -0.046 -0.0543 0.1087 0.1445 0.0119 0.945 0.091

ZBPD7 0.163 0.1395 0.1267 0.1501 0.0166 0.928 0.268

**ZBPD8** -0.448 -0.3642 0.1445 0.1837 0.0279 0.864 **0.876**

ZBPD9 -0.158 -0.1477 0.1021 0.1471 0.0105 0.948 0.320

MODEL RESULTS (bi-2 factor model)

ESTIMATES S. E. M. S. E. 95% % Sig

Population Average Std. Dev. Average Cover Coeff

FG BY

ZBPD1 0.556 0.4435 0.1677 0.3756 0.0408 0.849 0.816

ZBPD2 0.614 0.5074 0.0904 0.1747 0.0195 0.808 0.923

ZBPD3 0.393 0.3097 0.1264 0.3358 0.0229 0.905 0.632

ZBPD4 0.588 0.4839 0.1158 0.2363 0.0242 0.840 0.884

ZBPD5 0.596 0.4697 0.1139 0.2671 0.0289 0.818 0.843

ZBPD6 0.576 0.4969 0.1804 0.3388 0.0388 0.884 0.895

ZBPD7 0.269 0.2085 0.1223 0.3095 0.0186 0.918 0.452

ZBPD8 0.563 0.4134 0.1303 0.4271 0.0393 0.809 0.744

ZBPD9 0.670 0.5308 0.1456 0.3019 0.0406 0.723 0.891

F1 BY

ZBPD1 -0.329 -0.2947 0.2301 0.4067 0.0541 0.843 0.372

ZBPD2 -0.012 -0.0361 0.1497 0.3795 0.0230 0.913 0.081

ZBPD3 0.286 0.1900 0.1925 0.3347 0.0463 0.646 0.293

ZBPD4 0.289 0.2021 0.2070 0.4143 0.0504 0.782 0.238

ZBPD5 0.355 0.2508 0.2218 0.4167 0.0600 0.758 0.305

ZBPD6 0.027 -0.0052 0.1242 0.3580 0.0165 0.876 0.047

ZBPD7 -0.012 -0.0058 0.1244 0.2395 0.0155 0.853 0.067

ZBPD8 -0.482 -0.3961 0.2749 0.4408 0.0830 0.816 0.482

ZBPD9 -0.092 -0.0896 0.1451 0.3325 0.0210 0.712 0.106

F2 BY

ZBPD1 0.009 0.0120 0.1489 0.2764 0.0222 0.934 0.044

ZBPD2 0.019 -0.0183 0.1993 0.3544 0.0411 0.932 0.062

ZBPD3 -0.238 -0.2588 0.2674 0.3917 0.0719 0.753 0.281

ZBPD4 0.093 0.0088 0.1735 0.3364 0.0372 0.748 0.064

ZBPD5 -0.019 -0.0821 0.1753 0.3306 0.0347 0.854 0.103

ZBPD6 0.259 0.1986 0.2353 0.4067 0.0590 0.805 0.162

ZBPD7 -0.388 -0.3730 0.3163 0.4372 0.1003 0.857 0.382

ZBPD8 -0.010 0.0236 0.1281 0.2572 0.0175 0.903 0.044

ZBPD9 0.296 0.2015 0.2178 0.3785 0.0564 0.773 0.177

MODEL RESULTS (bi-3 factor model)

ESTIMATES S. E. M. S. E. 95% % Sig

Population Average Std. Dev. Average Cover Coeff

F1 BY

ZBPD1 0.500 0.4275 0.1058 0.2046 0.0164 0.923 0.**850**

ZBPD2 0.608 0.5216 0.0860 0.1530 0.0148 0.878 0.**957**

ZBPD3 0.390 0.3015 0.1111 0.2718 0.0202 0.902 0.639

ZBPD4 0.618 0.5022 0.1733 0.3335 0.0434 0.795 0**.927**

ZBPD5 0.632 0.4882 0.1529 0.3312 0.0441 0.765 **0.893**

ZBPD6 0.613 0.4946 0.1621 0.2967 0.0403 0.803 **0.928**

ZBPD7 0.284 0.2144 0.1078 0.1802 0.0165 0.921 0.436

ZBPD8 0.498 0.4147 0.1150 0.2391 0.0202 0.920 **0.785**

ZBPD9 0.671 0.5415 0.2103 0.3828 0.0610 0.778 **0.917**

F2 BY

ZBPD1 0.447 0.3791 0.3708 0.7278 0.1421 0.811 0.292

ZBPD2 0.096 0.0370 0.1272 0.2105 0.0197 0.858 0.024

ZBPD3 -0.042 -0.0506 0.1198 0.1635 0.0144 0.855 0.043

ZBPD4 -0.188 -0.1678 0.2700 0.4268 0.0733 0.762 0.089

ZBPD5 -0.227 -0.1824 0.2493 0.3394 0.0641 0.727 0.119

ZBPD6 -0.123 -0.0514 0.1266 0.1969 0.0212 0.709 0.016

ZBPD7 0.015 -0.0110 0.0895 0.1302 0.0087 0.932 0.015

ZBPD8 0.559 0.4715 0.4280 0.9957 0.1908 0.816 0.331

ZBPD9 0.113 0.0489 0.1093 0.1861 0.0161 0.668 0.042

F3 BY

ZBPD1 0.056 0.0133 0.1349 0.2059 0.0200 0.887 0.008

ZBPD2 -0.011 -0.0065 0.2158 0.3684 0.0466 0.975 0.019

ZBPD3 0.420 0.3941 0.4402 0.9171 0.1944 0.800 0.202

ZBPD4 0.048 0.0736 0.2270 0.3491 0.0522 0.909 0.053

ZBPD5 0.129 0.1498 0.3052 0.5525 0.0936 0.762 0.084

ZBPD6 -0.358 -0.3182 0.4771 1.3458 0.2292 0.714 0.073

ZBPD7 -0.001 0.0341 0.1113 0.1674 0.0136 0.970 0.029

ZBPD8 -0.031 -0.0360 0.1089 0.1945 0.0119 0.910 0.019

ZBPD9 -0.176 -0.1200 0.2314 0.3938 0.0567 0.674 0.046

F4 BY

ZBPD1 -0.060 -0.0497 0.1411 0.1540 0.0200 0.861 0.022

ZBPD2 0.068 0.1070 0.2855 0.4035 0.0830 0.955 0.031

ZBPD3 0.044 0.0800 0.1452 0.1627 0.0224 0.851 0.062

ZBPD4 -0.080 -0.0561 0.2402 0.3123 0.0583 0.895 0.019

ZBPD5 0.021 0.0611 0.1731 0.1911 0.0316 0.947 0.038

ZBPD6 0.029 -0.0016 0.1398 0.1760 0.0205 0.924 0.016

ZBPD7 0.606 0.4878 0.4915 1.4471 0.2555 0.729 0.223

ZBPD8 0.030 -0.0079 0.1092 0.1309 0.0134 0.915 0.014

ZBPD9 -0.243 -0.2711 0.3919 0.7564 0.1544 0.805 0.088

F1 WITH

F2 0.000 0.0000 0.0019 0.0001 0.0000 0.389 0.611

F3 0.000 0.0000 0.0018 0.0002 0.0000 0.470 0.530

F4 0.000 0.0001 0.0031 0.0001 0.0000 0.455 0.545

F2 WITH

F3 -0.295 -0.0832 0.1319 0.3615 0.0622 0.814 0.024

F4 -0.199 -0.0785 0.1363 0.3111 0.0331 0.919 0.019

F3 WITH

F4 0.234 0.0679 0.1311 0.3399 0.0448 0.890 0.013
